# Structural studies of human fission protein FIS1 reveal a dynamic region important for GTPase DRP1 recruitment and mitochondrial fission

**DOI:** 10.1016/j.jbc.2022.102620

**Published:** 2022-10-20

**Authors:** John M. Egner, Kelsey A. Nolden, Megan Cleland Harwig, Ryan P. Bonate, Jaime De Anda, Maxx H. Tessmer, Elizabeth L. Noey, Ugochukwu K. Ihenacho, Ziwen Liu, Francis C. Peterson, Gerard C.L. Wong, Michael E. Widlansky, R. Blake Hill

**Affiliations:** 1 Department of Biochemistry, Medical College of Wisconsin, Milwaukee, Wisconsin, USA; 2 Department of Bioengineering, University of California, Los Angeles, Los Angeles, California, USA; 3 Department of Microbiology & Immunology, Medical College of Wisconsin, Milwaukee, Wisconsin, USA; 4 Department of Biophysics, Medical College of Wisconsin, Milwaukee, Wisconsin, USA; 5 Department of Medicine, Medical College of Wisconsin, Milwaukee, Wisconsin, USA

**Keywords:** mitochondria, fission, protein dynamics, molecular dynamics, NMR, protein structure, intrinsic disordered region, tetratricopeptide repeat, hetNOE, heteronuclear NOE, MD, molecular dynamics, PDB, protein data bank, RMSF, root mean square fluctuation, RPE, retinal pigmented epithelial, TPR, tetratricopeptide repeat

## Abstract

Fission protein 1 (FIS1) and dynamin-related protein 1 (DRP1) were initially described as being evolutionarily conserved for mitochondrial fission, yet in humans the role of FIS1 in this process is unclear and disputed by many. In budding yeast where Fis1p helps to recruit the DRP1 ortholog from the cytoplasm to mitochondria for fission, an N-terminal “arm” of Fis1p is required for function. The yeast Fis1p arm interacts intramolecularly with a conserved tetratricopeptide repeat core and governs *in vitro* interactions with yeast DRP1. In human FIS1, NMR and X-ray structures show different arm conformations, but its importance for human DRP1 recruitment is unknown. Here, we use molecular dynamics simulations and comparisons to experimental NMR chemical shifts to show the human FIS1 arm can adopt an intramolecular conformation akin to that observed with yeast Fis1p. This finding is further supported through intrinsic tryptophan fluorescence and NMR experiments on human FIS1 with and without the arm. Using NMR, we observed the human FIS1 arm is also sensitive to environmental changes. We reveal the importance of these findings in cellular studies where removal of the FIS1 arm reduces DRP1 recruitment and mitochondrial fission similar to the yeast system. Moreover, we determined that expression of mitophagy adapter TBC1D15 can partially rescue arm-less FIS1 in a manner reminiscent of expression of the adapter Mdv1p in yeast. These findings point to conserved features of FIS1 important for its activity in mitochondrial morphology. More generally, other tetratricopeptide repeat–containing proteins are flanked by disordered arms/tails, suggesting possible common regulatory mechanisms.

Mitochondria continuously undergo fusion and fission to maintain their morphology, which is vital for maintenance of multiple cellular pathways including oxidative phosphorylation, calcium signaling, and stress-induced apoptosis (1, 2, 3, 4). Excess mitochondrial fission has been associated with several pathologies, including pulmonary arterial hypertension, ischemia-reperfusion injury, and diabetic cardiomyopathy (5, 6, 7, 8). Mitochondrial fission involves dynamin-related protein 1 (DRP1), which resides in the cytosol until recruited to mitochondria (9, 10). In mammals, DRP1 recruitment to mitochondria involves one of four mitochondrial outer membrane anchored recruiters: mitochondrial fission factor (MFF) (11, 12), mitochondrial dynamics protein of 49 kDa or 51 kDa (MID49, MID51) (13, 14), and FIS1 (15, 16, 17). Cell lines lacking individual, or a combination of, DRP1 recruiter proteins suggests each recruiter can uniquely support DRP1 recruitment (18, 19), and the nature of this recruitment is emerging (20, 21, 22, 23). Of the four DRP1 recruiters, FIS1 is the only recruiter conserved across all species containing mitochondria, suggesting a fundamental requirement for FIS1 (24).

FIS1 is a Type II integral membrane protein anchored to the mitochondrial outer membrane exposing an N-terminal domain comprised of two tetratricopeptide repeats (TPRs) to the cytoplasm (15, 16, 17, 25, 26, 27). TPRs are common protein–protein interaction domains (28, 29, 30) and crosslinking data show human FIS1 coimmunoprecipitates with DRP1 under certain conditions (16, 31, 32). However, FIS1 KO in HCT116 cells does not change mitochondrial morphology (12), questioning a role for FIS1 in fission (3, 33). Contrary to this, deleting or attenuating FIS1 in some cell types elongates mitochondria (16, 17, 34); also, overexpression of FIS1 in many cell types, including neurons, causes fragmentation and apoptosis (12, 15, 16, 17, 35, 36). This discrepancy may arise from tissue-dependent specificities and/or that each mitochondrial recruiter of DRP1 is responsible for activating fission in distinct cellular pathways. Recent studies support MFF acting as the predominant DRP1 recruiter in "housekeeping" fission for distributing organelles (12, 37), and FIS1 recruiting the GTPase-activating proteins TBC1D15 and 17 to mitochondria to limit autophagosome formation during mitophagy (38, 39). Thus, human FIS1 may have a more pronounced role in stress-induced mitochondrial fission and mitophagy (32, 39, 40, 41, 42, 43, 44). For example, mouse embryonic fibroblasts lacking FIS1 retain ∼50% more cytochrome c upon apoptosis induction (37). In other stress-induced conditions, such as hypo- or hyper-glycemic stress associated with diabetes, FIS1 may act to recruit DRP1 culminating in excessive mitochondrial fission (8). Indeed, super resolution microscopy studies support that DRP1-dependent fission can involve either MFF for distribution of healthy mitochondria or FIS1 for removal of damaged mitochondria (45).

By contrast to the human system, budding yeast Fis1p is unequivocally involved in DRP1-mediated fission (Dnm1p in yeast) *via* the fungal-specific adapter protein Mdv1p (46, 47, 48, 49). Curiously, highly conserved residues in yeast Fis1p (Arg77, Tyr82, Ile85, Ly89) mediate Dnm1p binding in pull-down experiments. These residues are not in TPR consensus positions that specify the protein fold, suggesting that FIS1 may be conserved for DRP1 interactions (50). However, these residues in yeast Fis1p are normally occluded by an intramolecular interaction between 16 N-terminal residues (dubbed the FIS1 arm, Fig. 1) (51, 52). Deletion of the Fis1p arm in yeast abolishes Dnm1p recruitment and fission (51, 53). *In vitro*, the Fis1p arm negatively regulates Dnm1p binding, suggesting an autoinhibitory role (50). Whether the FIS1 arm is important in mammalian fission—where it is only eight residues long—is not known.

**Figure 1:**
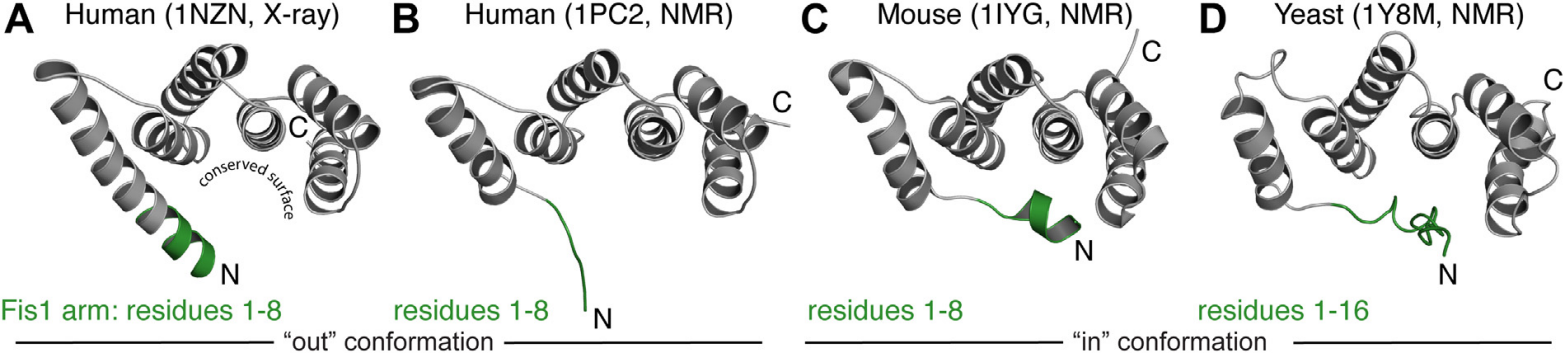
**Conformations of the FIS1 arm differ.** Ribbon representations of FIS1 cytoplasmic domain structures from (*A*) human by X-ray crystallography at 2.0 Å (1NZN.pdb), (*B*) human by NMR (1PC2.pdb), (*C*) mouse by NMR (1IYG.pdb), and (*D*) yeast by NMR (1Y8M.pdb). The FIS1 arm is highlighted in *green* and is comprised of residues 1 to 8, except in yeast FIS1 where it is eight residues longer. Human and mouse FIS1 sequences share 96% identity, with identical FIS1 arm sequences. Human and yeast FIS1 sequences share 28% sequence identity. Native FIS1 is 152 residues with a C-terminal transmembrane domain spanning 126 to 152. Constructs used to solve each structure differ with respect to length and presence of cloning artifacts (see S1). Disordered C-terminal residues in (*B*–*D*) and non-native N-terminal residues (GSSGSSG) from (*C*) (1IYG) were removed for clarity.

The mouse and human FIS1 arm adopt different conformations where the mouse NMR structures adopts a yeast-like intramolecular conformation that might occlude access to a conserved surface (Protein Data Bank [PDB] ID: 1IYG(29), Fig. 1). However, an N-terminal cloning artifact might be responsible for this conformation. In contrast to yeast and mouse, the human FIS1 arm is either disordered by NMR (PDB ID: 1PC2(27)) or helical by X-ray (PDB ID: 1NZN(28)). In either structure, the FIS1 arm does not adopt an intramolecular conformation and is flexible, as determined by NMR T_2_ measurements (26), or adopts a helical conformation that is stabilized by crystallographic lattice contacts, suggesting a crystal-induced artifact (27). Here, we report that the FIS1 arm can adopt a yeast-like “in” conformation and find that deletion of the arm impairs DRP1 recruitment to mitochondria and mitochondrial fission in a manner akin to the yeast system. Conversely, removal of the arm does not impact TBC1D15 mitochondrial recruitment. Strikingly, overexpression of TBC1D15 partially rescues the impaired mitochondrial fission activity of FIS1ΔN. These data support evolutionarily conserved features of FIS1 that are central to mitochondrial morphology in a DRP1-dependent manner.

## Results

### Human FIS1 NMR chemical shifts at physiological pH differ from OUT conformation

**Figure 2:**
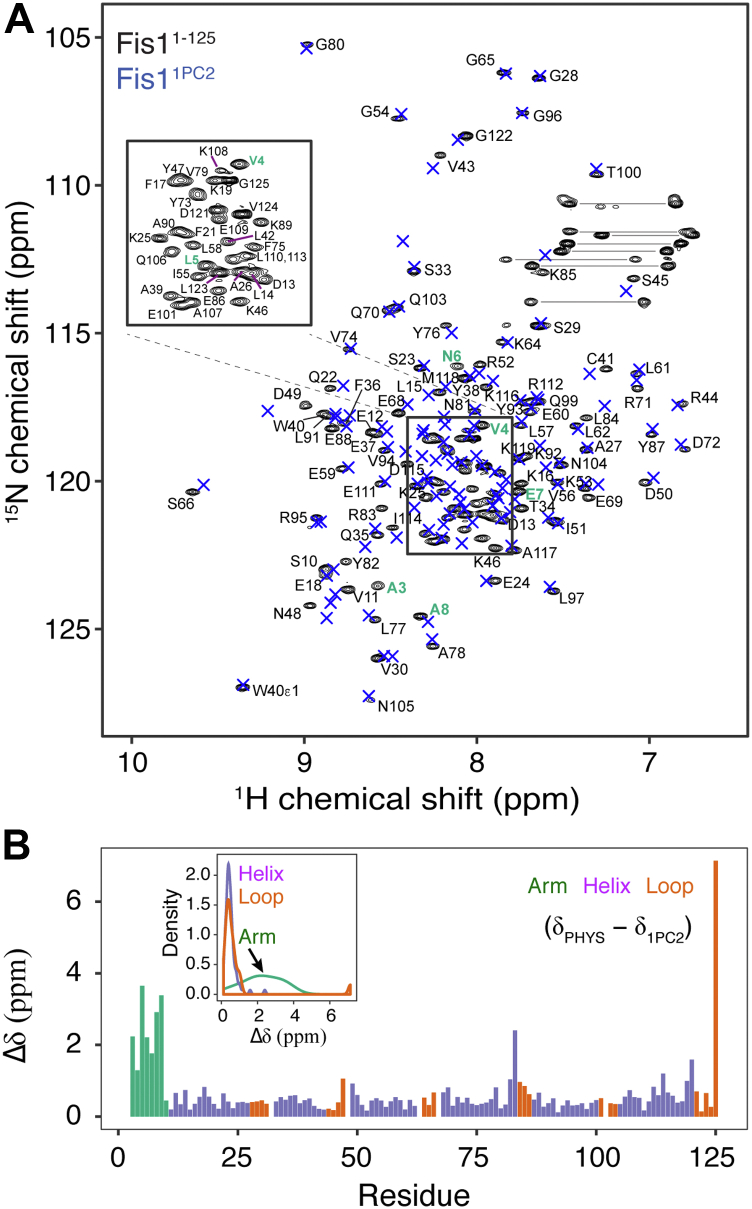
**Comparison of NMR chemical shifts between different constructs and conditions.***A*, ^1^H, ^15^N HSQC spectrum of 300 μM FIS1^PHYS^ at physiological pH; arm residues are colored *green* with M1 and E2 not detected. The chemical shifts of the FIS1 NMR structure 1PC2.pdb (FIS1^1PC2^) are indicated by × (*B*) The ^1^H/^15^N chemical shift differences were visualized by residue and secondary structure using a probability density distribution plot (inset), which shows distributions of chemical shifts between samples. Sample conditions for PHYS: residues 1 to 125, 100 mM Hepes pH 7.4, 200 mM NaCl, 1 mM DTT, 0.02% (w/v) sodium azide, 10% D_2_O, 298 K and 1PC2: residues 1 to 145 with 146 to 152 replaced with EHHHHHH, 10 mM Tris Acetate pH 5.5, 10% D_2_O, 305 K. Residues are colored based on secondary structure as indicated.

The structural differences in the orientation of the FIS1 arm between the human and mouse NMR structures (Fig. 1, *B* and *C*) are curious, given that the sequences only differ by six residues, four of which are conservative substitutions and none of which are proximal to the arm (Fig. S1). Despite this, the mouse FIS1 sequence adopts an intramolecular or IN conformation with respect to the FIS1 arm, while the human arm is exposed in solution (referred to here as an OUT conformation). These structural differences may arise simply from differences in the constructs and conditions used in structure determination as they vary in sequence length, presence of cloning artifacts, and buffer conditions, neither of which was close to physiological pH. Given this and the known pH sensitivity of the yeast FIS1 arm (54, 55), we asked whether pH might influence the FIS1 conformation. For this, human FIS1^1-125^ was uniformly labeled with ^15^N and ^1^H/^15^N chemical shifts were recorded at physiological pH—referred to here as FIS1^PHYS^—and compared to those previously published from the solution structure of FIS1^1–152^ (1PC2.pdb), referred to here as FIS1^OUT^. Chemical shifts differ throughout the spectral overlay (Fig. 2*A*) with the most significant perturbations in the FIS1 arm and at the C terminus (Fig. 2*B*); the latter being expected given the extra 27 residues in the 1PC2/FIS1^OUT^ construct. These differences were visualized using kernel density plots based on secondary structural elements (Fig. 2*B* inset), which provides an effective means of determining whether the distributions of chemical shifts between samples are significant. Chemical shifts in the helical and loop regions are distributed as expected for random differences between samples. By contrast, the chemical shift distribution for FIS1 arm residues is skewed, indicating a larger difference between FIS1^PHYS^ and FIS1^OUT^ than might be expected solely from sample conditions. These chemical shift differences could arise from differences either in arm conformation or in sample conditions. To differentiate this, we recorded ^1^H/^15^N heteronuclear single quantum coherence (HSQC) spectra on FIS1^1–125^ with identical buffer and temperature conditions used previously in solving the solution structures of mouse and human proteins. Kernel density plot analyses of these data showed randomly distributed changes (Fig. S2), indicating the FIS1 arm differences in Fig. 2 arise from differences in the constructs and not pH, temperature, or buffer.

### Molecular dynamics simulations reveal FIS1 arm is dynamic and may adopt IN conformation

**Figure 3:**
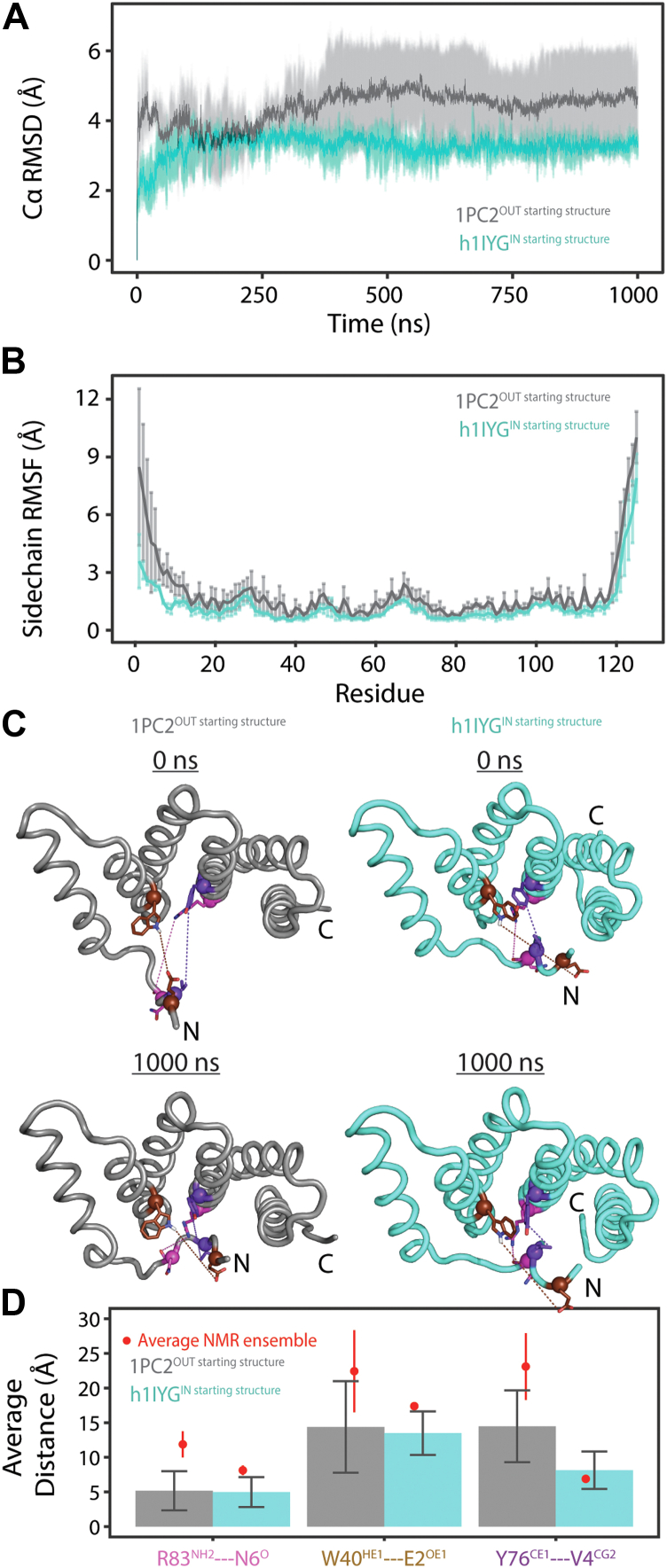
**The human FIS1 arm adopts an IN conformation in 1 μs molecular dynamics (MD) simulations regardless of starting structure.***A*, the average C_α_ RMSD and SD is shown from three 1000 ns MD replicates with the indicated starting structures of FIS1 using GROMACS v2018 with the Amber99SB force field and TIP3P water model with 140 mM KCl charge neutralization in a dodecahedron box, which extended >10 Å from the edge. *B*, the average root mean square fluctuation (RMSF) and SD, a measure of sidechain flexibility, for each FIS1 residue across all 1000 ns trajectories shown in panel (*A*). *C*, representative initial (0 ns) and final (1000 ns) FIS1 conformations are shown for each starting structure. Colored spheres indicate atom–atom distances measured between three different pairs of residues, where each pair is comprised of an atom from the FIS1 TPR core or arm. *D*, comparison of average distances shown in (*C*) calculated over the entire trajectory (bars) relative to the average distances (*red circle*) and SDs (*vertical red line*) calculated from the 20 deposited structures of human (1PC2) or mouse (1IYG) FIS1 (offset for clarity). The selected atoms are representative of short, medium, and long-range distances in both 1IYG and final simulations. Note that SDs for 1IYG were less than the size of the *red circle*. Data are presented as mean ± SD, n = 3. Atom–atom distances between starting structures are not significant by an ANOVA.

**Figure 4:**
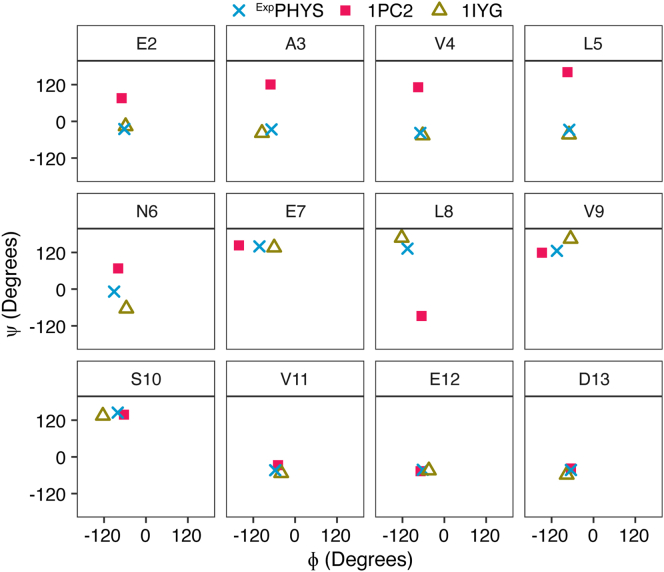
**Talos+ torsion angle predictions between experimental FIS1 chemical shifts and FIS1 structures suggest the arm adopts a helical conformation similar to mouse *FIS1*.** The backbone torsion angles for FIS1 arm residues were estimated using Talos+ (52) from NMR chemical shifts at physiological pH reported here (FIS1^PHYS^, × ), solution structure of hFIS1 (1PC2, ▪), and solution structure of mFIS1 isoform 1 (1IYG, **▵**).

To evaluate possible conformations of the FIS1 arm, we sampled FIS1 conformational space with 1000–ns molecular dynamics (MD) simulations. To also assess whether the MD simulations were influenced by the starting structure, we performed MD simulations using two starting structures; the solution structure of FIS1 (PDB ID: 1PC2) and a homology model of FIS1 derived from the solution structure of mouse FIS1 isoform 1 (referred to as h1IYG, Fig. 3, Movies S1 and S2). For all simulations, the Cα RMSD values rapidly increased initially (0–100 ns) and leveled off by ∼200 ns (Fig. 3*A*, S3*A*). Trajectories with starting structure 1PC2 have greater root mean square fluctuation (RMSF) values of FIS1 arm residues than trajectories with starting structure h1IYG, where the FIS1 arm remains in an arm IN conformation throughout all trajectories (Fig. 3*B*, S3*B*). The higher RMSF values and overall extended arm conformation of 1PC2 likely explains the greater RMSD values of trajectories with this starting structure (1PC2) compared to h1IYG. Representative images of simulation snapshots at 0 and 1000 ns using starting structures 1PC2 and h1IYG are shown in Fig. 3*C*. Regardless of starting structure, the FIS1 arm adopts an IN conformation through intramolecular contacts with the FIS1 conserved surface (Fig. 3*C*, S3). We used sidechain atom–atom distances between residues residing in the arm and TPR core of FIS1 to infer and quantify the arm IN/OUT conformations. For this, we chose atoms that were representative of short, medium, and long distances in the mouse 1IYG structure: R83^NH2^:N6^O^, W40^HE1^:E7^OE1^, and Y76^CE1^:V4^CG2^ (Figs. 3*C*, 4D, S3*C*). For comparison, these same atom–atom distances were measured and averaged across each 20-state ensemble of previously solved solution structures of FIS1 (PDB ID: 1PC2) and mouse FIS1 (PDB ID: 1IYG, shown in Fig. 3*D* as *red circles*). As reflected visually (Fig. 3*C*), all atom–atom distances for starting structure 1PC2 trajectories were less than the ensemble reference. In addition, distances were close or in identical agreement with the starting structure h1IYG trajectories, indicating the FIS1 arm adopts an IN conformation regardless of starting structural conformation. Interestingly, each trajectory had R83 and N6 being 4 Å or less apart from one another, suggesting a potential favorable hydrogen bonding interaction comprising the arm IN conformation, which is typical for specifying a disorder-to-order conformation (56). These data and Sparta+ NMR chemical shift predictions based on MD simulations (Fig. S4) support the possibility that the human FIS1 arm might be similar to the yeast and mouse FIS1 homologs in being able to adopt an IN conformation.

### NMR derived torsion angles for FIS1 arm

We next asked if the NMR backbone torsion angle data supported a FIS1 arm IN conformation. In the mouse FIS1 NMR structure (1IYG), residues 2 to 6 of the arm form a small helix with expected backbone torsion angles for φ and ψ. FIS1 arm torsion angles were determined from HN, HA, CA, CB, CO, and N chemical shifts using Talos+ (57) and compared to the published FIS1 structures representing an arm IN (PDB ID: 1IYG) and OUT (PDB ID: 1PC2) conformation (Fig. 4). The experimentally derived values for FIS1 residues 2 to 5 lie in practically identical Ramachandran space as the arm IN conformation (1IYG). Residues 7 to 13 are in similar Ramachandran space throughout all three molecules, which is expected since little to no difference between IN and OUT conformations exists for those residues (see Fig. 1). These data suggest that under physiological conditions the FIS1 arm might adopt a small helix consistent with the arm forming intramolecular contacts similar to mouse FIS1.

### FIS1 arm is sensitive to environmental conditions based on NMR spin relaxation experiments

**Figure 5:**
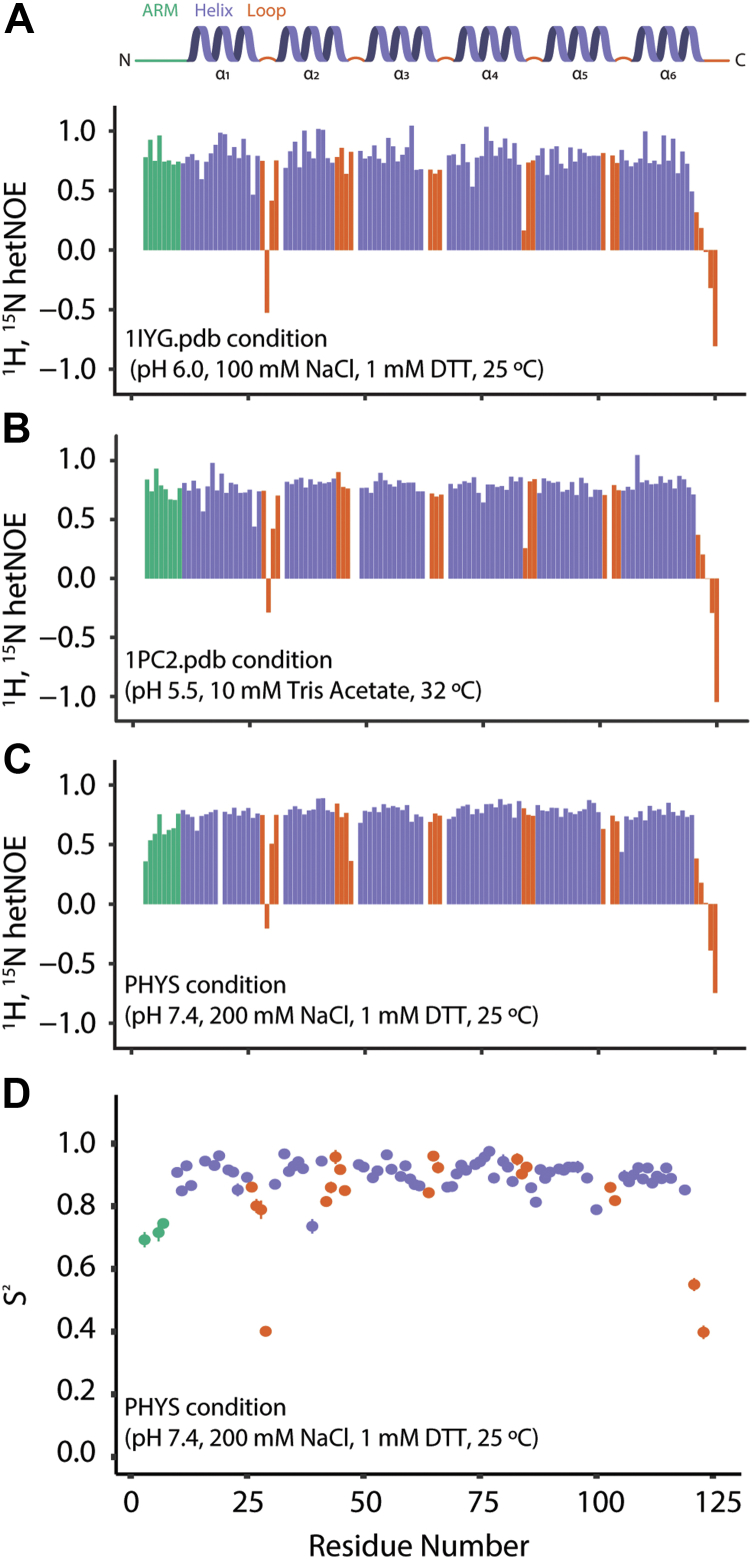
**NMR spin relaxation analyses of FIS1 under different conditions*.***^1^H, ^15^N, heteronuclear-NOE (het-NOE) plots of ^15^N-FIS1^1-125^ in (*A*) IN, (*B*) OUT, and (*C*) physiological pH sample conditions. *D*, generalized order parameter S^2^ calculated from R_1_, R_2_, and het-NOE NMR spin relaxation measurements at 11.7 and 14.1 T using the Lipari–Szabo model-free formalism. Residues are colored by secondary structure as in Fig 2*B*. For (*A*–*C*), het-NOE data at 14.1 T were collected on 300 μM ^15^N-FIS1^1-125^ in the following conditions; physiological (PHYS): 100 mM Hepes pH 7.4, 200 mM NaCl, 1 mM DTT, 0.02% sodium azide, 10% D_2_O, 25 °C; OUT (FIS1^1PC2^) condition: 10 mM Tris acetate pH 5.5, 10% D_2_O, 32 °C; and IN (mFIS1^1IYG^) condition: 20 mM sodium phosphate pH 6.0, 100 mM NaCl, 1 mM DTT, 10% D_2_O, 25 °C. For (*D*), data were collected on 600 μM ^15^N-FIS1^1-125^ at 298K under PHYS conditions.

The backbone dynamics of the FIS1 arm might be sensitive to sample conditions and were evaluated using ^1^H, ^15^N heteronuclear NOE (hetNOE) NMR spectroscopy. The hetNOE is sensitive to backbone dynamics on the ps–ns timescale with values of ∼0.8 for structured regions and much lower values for unstructured regions (58). We first tested FIS1^1-125^ under the 1IYG sample conditions (*i.e*., IN condition). As expected for a structured region, FIS1 arm residues had average hetNOE values of 0.8 ± 0.1, consistent with a structured IN conformation (Fig. 5*A*). Unexpectedly, upon changing sample conditions to those found for the reference FIS1 structure (1PC2) with the arm OUT, we did not observe a large decrease in hetNOE values in the FIS1 arm with an average value of 0.77 ± 0.09 (Fig. 5*B*), indicating that arm is not disordered. This unexpected finding indicates that the FIS1 arm is sensitive to the length of the construct, which differs between the prior and present studies by 27 C-terminal residues. Under physiological pH 7.4 (PHYS), the FIS1 arm hetNOE values were the lowest of all three conditions with an average value of 0.6 ± 0.13 (Fig. 5*C*). Additional hetNOE, along with R_1_ and R_2_, spin relaxation data were collected under PHYS conditions at two magnetic field strengths (Fig. S5) and analyzed using the model-free formalism to determine per residue generalized order parameters, S^2^ (Fig. 5*D*). Helical regions of FIS1 give an average S^2^ = 0.90 ± 0.04 typical of well-structured helical proteins. FIS1 arm order parameters are lower (S^2^ = 0.72 ± 0.04) but do not approach the values found for disordered N and C termini that are typically 0.5 or lower. We interpret these data to indicate that the FIS1 arm is capable of sampling different conformational states, a subset of which might adopt an IN conformation but likely with dynamics that are sensitive to environmental conditions.

### Arm deletion impacts residues in TPR core

**Figure 6:**
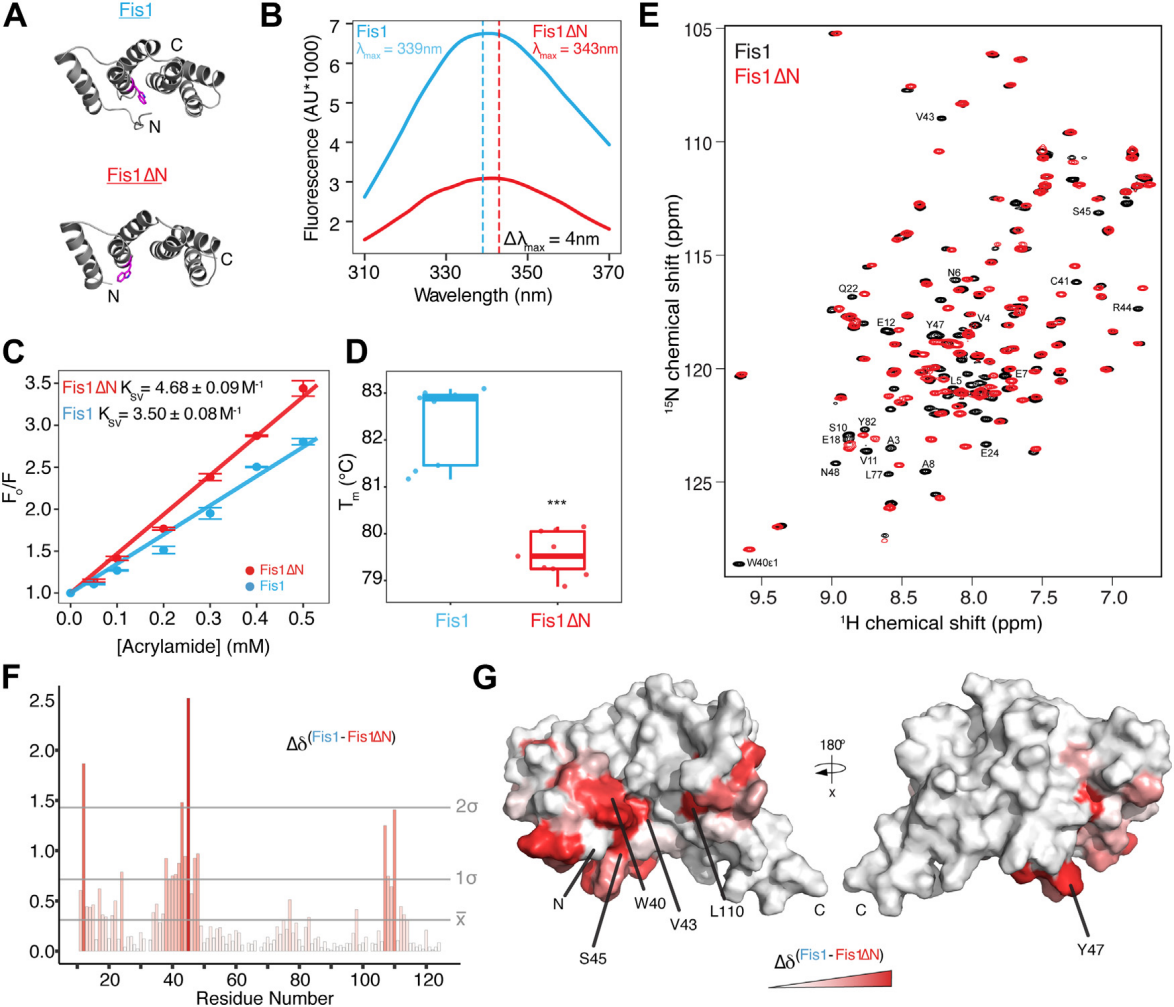
**The FIS1 arm occludes residue W40 within the TPR core and forms intramolecular contacts with TPR core residues.***A*, ribbon representations of FIS1 (1PC2) and a model of FIS1 lacking the FIS1 arm (FIS1ΔN) showing the location of W40 (*magenta sidechain*). *B*, tryptophan emission spectra for FIS1 (*blue line*) and FIS1ΔN (*red line*) were collected on 10 μM samples (λ_ex_ = 295 nm). The maximum wavelength (λ_max_) is depicted by a dashed vertical line for each FIS1 construct. Spectra are representative of three biological replicates. Sample buffer comprised of 100 mM Hepes pH 7.4, 200 mM NaCl, 1 mM DTT, and 0.02% (w/v) sodium azide. *C*, fluorescence of FIS1 (*blue line*) and FIS1ΔN (*red line*) at 341 nm alone divided by the fluorescence in the presence of increasing concentrations of the quenching agent acrylamide (F_0_/F) ± SD. The Stern–Volmer constant ± SD was then calculated for each construct. Sample conditions as in (*B*) and represent three technical replicates. *D*, box and whiskers plot depicting the melting temperature of FIS1 (*blue*) and FIS1ΔN (*red*). *T*_m_ determined as the temperature corresponding to the first derivative of the maximum fluorescence value. Data are representative of three biological replicates, each with three technical replicates. ∗∗∗ *p* < 0.03 *E*, ^1^H, ^15^N HSQC spectral overlays of FIS1 and FIS1 lacking the arm (FIS1ΔN). *F*, chemical shift perturbations (Δδ) are shown for each residue between FIS1 and FIS1ΔN in a gradient fashion, where a *redder color* indicates a greater Δδ. Lines indicate one and two SDs. *G*, FIS1 residue Δδ are displayed on the surface representation of FIS1^1PC2^ (with the arm removed for clarity) in a gradient fashion, replicating the coloring scheme in panel (*F*). The right surface representation depicts FIS1 rotated about the *x*-axis by 180° to display the convex face of FIS1.

To further evaluate the arm conformation, we turned to fluorescence spectroscopy. FIS1 has a single tryptophan (W40) located on helix 2 of the concave surface, which we reasoned might serve as a label-free reporter of the FIS1 arm conformation. If the FIS1 arm adopts an IN conformation, W40 might be occluded by the arm and less solvent exposed (Fig. 6*A*, top panel), resulting in a fluorescence intensity increase and λ_max_ decrease when compared to a FIS1 arm OUT conformation or against a FIS1 variant lacking the arm (FIS1ΔN, Fig. 6*A*, bottom panel). To assess this, intrinsic tryptophan fluorescence emission spectra were collected on FIS1^PHYS^ and FIS1ΔN (Fig. 6*B*). Upon deletion of the FIS1 arm, FIS1ΔN fluorescence intensity decreased by approximately 4000 AU with an increase in λ_max_ by 4 nm, consistent with W40 being more solvent exposed upon arm deletion. If true, then FIS1 arm deletion should allow more efficient collisional quenching of the W40 fluorescence signal. Using the quenching agent acrylamide, the Stern–Volmer coefficient significantly increased from 3.50 ± 0.08 M^-1^ to 4.68 ± 0.09 M^-1^ upon arm deletion (Fig. 6, *C* and *D*). These data indicate that in the presence of the arm, W40 is less solvent exposed and strongly support that the FIS1 arm can form intramolecular contacts with the concave surface where W40 resides. Next, we evaluated the effects of removing the FIS1 arm on protein thermal unfolding using differential scanning fluorimetry (Fig. 6*D*). The midpoint of the unfolding transition, *T*_m_, decreased ∼3 °C from 82.4 ± 0.8 °C to 79.6 ± 0.5 °C upon FIS1 arm deletion. This could arise from loss of stabilizing intramolecular interactions from the N-terminal eight residues and either helix 1 to cause helix fraying or the FIS1 concave surface. Given the NMR backbone chemical shift data indicating that residues 1 to 10 are nonhelical and the acrylamide fluorescence quenching data indicating increased solvent accessibility upon arm deletion, we interpret the thermal unfolding data to support that an arm IN conformation is possible.

If the FIS1 arm appreciably populates an IN conformation, then chemical shift perturbations in the TPR core upon arm deletion would also be observed. To this end, we collected ^1^H, ^15^N HSQC spectra on ^15^N-labeled FIS1^1-125^ and FIS1ΔN^9-125^ and computed total ^1^H, ^15^N chemical shift perturbations between each residue (Fig. 6, *E* and *F*). In agreement with results from fluorescence experiments, W40 experienced a statistically significant chemical shift perturbation of 0.75 ppm upon arm deletion. Additionally, chemical shifts of 10 residues were perturbed greater than two SDs from the mean; highlighting these perturbations on a surface representation of FIS1^1PC2^ indicates that two regions in the TPR core change significantly upon arm removal involving residues on helix 2 (Val43, Arg44, Ser45) and helix 6 (Ala107, Leu110) (Fig. 6*G*). These chemical shift perturbations lie in the TPR core in similar regions of Fis1 that also mediate arm-core interactions in the yeast and mouse structures. We interpret the collective biophysical data to indicate that the arm can form intramolecular contacts with the conserved surface of FIS1 with an ability to adopt both IN and OUT conformations depending on conditions.

### FIS1 arm is required for FIS1 activity

**Figure 7:**
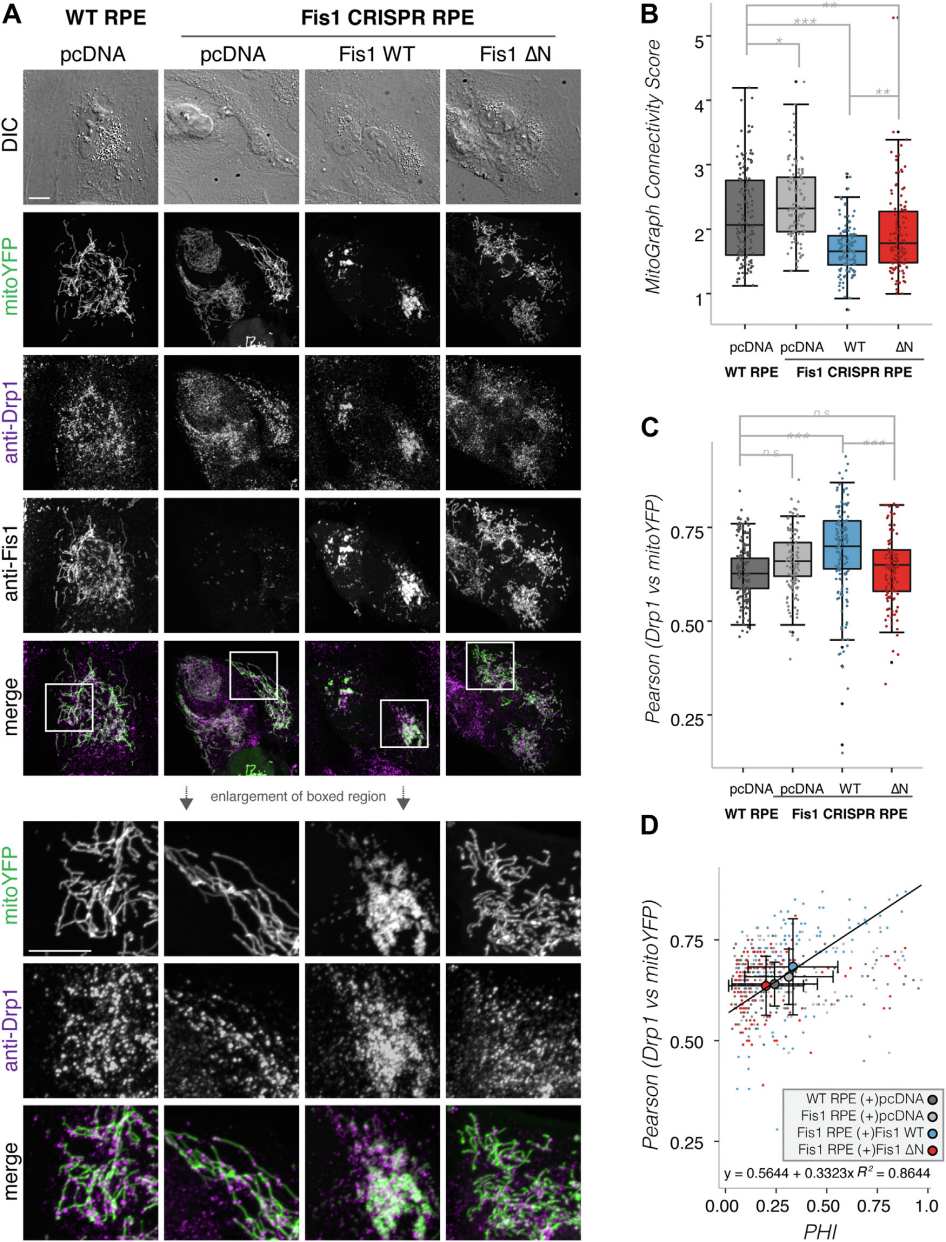
**Overexpression of WT FIS1, but not ΔN, induces accumulation of DRP1 and fragmentation/clumping of the mitochondrial network*.****A*–*D*, WT or FIS1 CRISPR KO RPE cells were transfected with mitoYFP and either pcDNA (*gray*), pcDNA-FIS1 WT (*blue*), or pcDNA-FIS1ΔN (*red*), fixed and immunostained sequentially for DRP1, followed by FIS1. *A*, representative confocal images of anti-FIS1, mitoYFP, and anti-DRP1. Merged images show DRP1 (magenta) and mitochondrial localization (mitoYFP; green). *B*, single cell z-stack images of mitoYFP transfected cells were segmented by MitoGraph and the resulting MitoGraph Connectivity score for mitoYFP was calculated by taking the ratio of profission and profusion MitoGraph metrics (see experimental procedures and/or Fig. S10 for details). *C*, the colocalization between mitoYFP and DRP1 from the same single cell z-stack images as in (*B*) was measured using Pearson’s Correlation R value. *D*, correlation plot between Pearson’s R value and MitoGraph PHI Score, which measures the fraction of mitochondria in the largest connected component (see text) and increases for the clumped or elongated/interconnected morphologies. Small dots are single cells; while large circles are population means. The trend line was calculated using the population means. *p*-values were calculated by ANOVA followed by TUKEY post hoc analysis; *p*-values: ∗ (*p* < 0.05); ∗∗ (*p* < 0.01); ∗∗∗ (*p* < 0.001). n.s. = not significant. The scale bar represents 10 microns. RPE, retinal pigmented epithelial.

In budding yeast, the FIS1 arm can also adopt an IN conformation, which is required for FIS1 activity (51, 53, 55, 59). Based on this, we asked whether the FIS1 arm is also required for human FIS1 activity. To test this, we first removed the FIS1 gene using CRISPR/Cas9 technology from human retinal pigmented epithelial (RPE) cells, known for robust mitochondrial respiration, by targeting two nickase pairs positioned on exon 4 of FIS1 using a Cas9n (D10A nickase mutant) (Fig. S6*A*). We isolated clonal populations and verified complete KO of FIS1 by Western blot (Fig. S6*B*). Transfection of these or WT RPE cells with mitoYFP and pcDNA-FIS1 (WT or ΔN) allowed visualization of the effects of FIS1 on mitochondrial morphology (Fig. 7). Mitochondria adopt a complex morphology with elongated and branched, but also more punctiform, structures within a single cell. FIS1 overexpression substantially fragmented the mitochondrial network (Fig. 7*A*). In addition to fragmentation, the mitochondria appeared clustered together upon FIS1 overexpression in an oftentimes perinuclear manner in agreement with previous observations (12, 15, 16, 17, 60). We refer to this clustering as a “clumped” morphology. Notably, these effects were weakened upon overexpression of FIS1ΔN (Fig. 7*A*), indicating that the FIS1 arm is important for fragmentation and clumping of the mitochondrial network. These effects were also observed upon overexpressing FIS1 and FIS1ΔN in a second RPE FIS1 CRISPR cell line (generated using the second nickase pair). We verified that this weakened phenotype was not specific to these epithelial cells as we found similar results in an endothelial cell line (HMEC-1) and in HeLa cells (Figs. S7–S9).

Mitochondrial morphology was quantified using MitoGraph v3.0 (34, 61), which is an open-source fully automated C++ program that generates a 3D surface model of the mitochondrial network from 3D confocal microscopy images (Fig. S10). MitoGraph output contains raw numbered node to node distances, which represent distances between mitochondrial end points and/or branch points. Graph theory was used to extract a variety of metrics based on these node to node distances using R. Briefly (see Experimental procedures for more detailed explanations), these metrics include *PHI* (fraction of total mitochondria occupied by a single, large mitochondrion), average edge length (distance between branch points or length of individual mitochondrion), nodes (number of branch points or end points), edges (number of branches or individual mitochondrion), connected components (number of connected mitochondria in a cell), and average degree (based on nearest neighbor analysis identifying free ends and branch points). These morphometric parameters can be combined into the MitoGraph connectivity score, which sums parameters elevated in highly fused networks (PHI, average edge length and average degree) and divides this value by the sum of parameters elevated in highly fragmented networks (total nodes, edges, and connected components). Visual examination of the confocal images of WT *versus* FIS1 CRISPR RPE cells reveals both conditions have elongated mitochondria characteristic of normal healthy cells. Despite the visual similarity, MitoGraph analysis revealed a modest, but statistically significant increase in the MitoGraph connectivity score upon FIS1 deletion (Fig. 7, *A* and *B*). Cells overexpressing FIS1 WT resulted in a significant decrease in the MitoGraph connectivity score reflecting increased fragmentation, whereas cells overexpressing FIS1ΔN had a higher MitoGraph connectivity score in agreement with the visual observation of impaired mitochondrial fragmentation (Fig. 7, *A* and *B*). Strikingly, the clumping of mitochondria observed upon expression of FIS1 WT was lost upon arm deletion. Intriguingly PHI, which reports on the portion occupied by the largest connected component is normally reduced in a highly fragmented network. However, we observed elevated PHI values for FIS1 WT but not ΔN (Fig. S11). We hypothesize that these elevated PHI values are reporting on the highly clumped mitochondria present in a large portion of the cells we imaged for FIS1 WT but not ΔN. Other metrics typically found to be elevated in a highly fragmented state (total edges and nodes) were elevated in FIS1 WT and less so in ΔN, while metrics typically associated with a highly fused state such average edge length were substantially reduced in FIS1 WT and less so in ΔN (Fig. S11). Some of the metrics indicated a slight increase in fission activity of FIS1ΔN (reduction in three-way junctions and increase in the number of free ends) (Fig. S11). In summary, FIS1 WT expression drove clumping and reduced the length and number of branch points of mitochondria while increasing the total number of individual mitochondria. By contrast, FIS1ΔN expression appeared to reduce branching but had less impact on the length of mitochondria with little clumping.

To evaluate if these changes were due to expression level differences, we quantified the mean cellular intensity of FIS1 and DRP1 signals from the confocal images used for MitoGraph analysis. We also evaluated immunoblots for FIS1 and DRP1 protein expression on lysates from WT or FIS1 CRISPR RPE cells expressing vector, WT and FIS1ΔN. For DRP1, fluctuations in the mean cellular immunofluorescence intensity indicated little changes in DRP1 intensity upon FIS1 deletion or expression of WT or FIS1ΔN (Fig. S12, *A*, *B* and *D*). This was confirmed by Western blot analysis demonstrating little to no change in DRP1 protein levels (Fig. S12, *A* and *B*). For FIS1, both Western blot and image analysis (Fig. S12*C*) revealed that FIS1 WT was expressed nearly 8-fold higher than endogenous levels and nearly 6-fold higher than FIS1ΔN. Thus, FIS1ΔN expression was more similar to endogenous FIS1 than overexpressed FIS1 (Fig. S12, *A*–*D*). Regardless, the morphological differences between FIS1 WT and ΔN were independent of the decreased expression, as limiting the quantification to only cells that express similar amounts of protein (noted by shaded *gray* areas in Fig. S12, *E* and *F*) gave similar results with FIS1ΔN expression, resulting in impaired fission and no clumping (Fig. S12, *G* and *H*). Thus, deletion of FIS1 arm reduces activity.

We assessed whether the increased fragmentation and clumping upon FIS1 expression correlated with increased DRP1 localization to mitochondria. Overexpression of WT, but not FIS1ΔN, led to an accumulation of endogenous DRP1 on mitochondria (Fig. 7*A*), which was most evident in the highly clumped mitochondria. This enhanced mitochondrial localization of DRP1 resulted in a statistically significant increase in the Pearson’s correlation R value between DRP1 and mitoYFP in cells expressing WT but not FIS1ΔN (Fig. 7*C*). The MitoGraph PHI score was well correlated with mitochondrial localization of DRP1 (R^2^ = 0.86, Fig. 7*D*), suggesting that the clumping phenotype is DRP1 and FIS1 arm dependent. Thus, these data support the FIS1 arm is important for FIS1 activity in fragmenting and clumping mitochondria, a process that likely involves the recruitment of DRP1 and is enhanced by the FIS1 arm.

#### TBC1D15 mitochondrial localization does not require FIS1 N-terminal arm, but coexpression can partially the rescue ΔN phenotype

**Figure 8:**
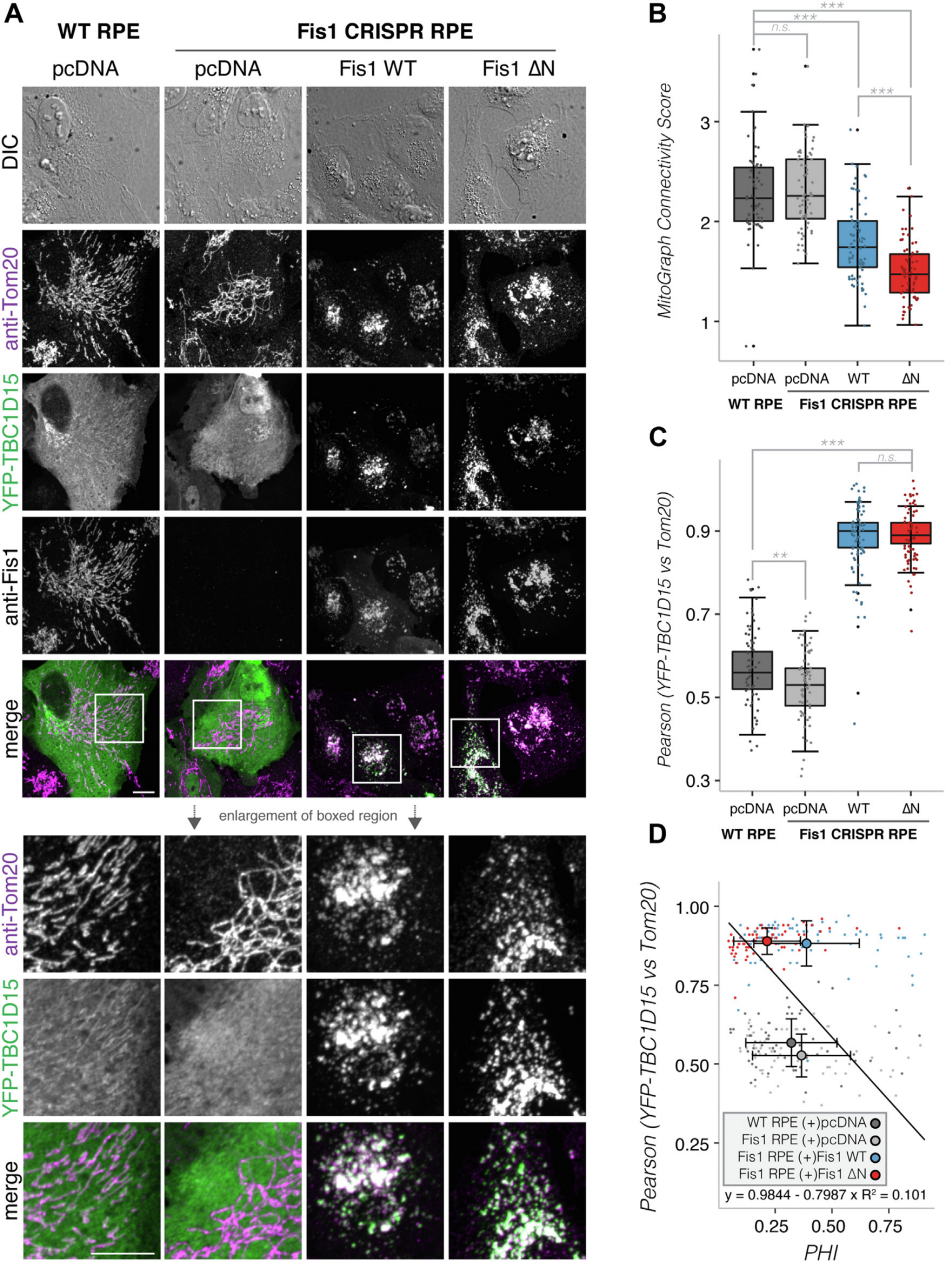
**TheFIS1armis not required for mitochondrial recruitment of TBC1D15*.*** ,*A*–*D* WT or FIS1 CRISPR KO RPE cells were transfected with YFP-TBC1D15 and either pcDNA (*gray*), pcDNA-FIS1 (*blue*), or pcDNA-FIS1ΔN (*red*), fixed and immunostained sequentially for Tom20, followed by FIS1. *A*, representative confocal images of anti-FIS1, YFP-TBC1D15, and anti-Tom20. Merged images show YFP-TBC1D15 (*green*) localization to mitochondria (Tom20; *magenta*). *B*, single cell z-stack images of YFP-TBC1D15 transfected cells were segmented by MitoGraph and the resulting MitoGraph Connectivity score for Tom20 segmentation was calculated by taking the ratio of profission and profusion MitoGraph metrics (see experimental procedures and/or Fig S12 for details). *C*, the colocalization between YFP-TBC1D15 and Tom20 from the same single cell z-stack images as in (*B*) was measured using Pearson’s Correlation R value. *D*, correlation plot between Pearson’s R value and MitoGraph PHI Score, which measures the fraction of mitochondria in the largest connected component (see text) and increases for the clumped or elongated/interconnected morphologies. *p*-values were calculated by ANOVA followed by TUKEY post hoc analysis; *p*-values: ∗ (*p* < 0.05); ∗∗ (*p* < 0.01); ∗∗∗ (*p* < 0.001). n.s. = not significant. The scale bar represents 10 microns. RPE, retinal pigmented epithelial.

In addition to interactions with DRP1, FIS1 has recently been shown to interact with the mitophagy adapter TBC1D15(42). To examine if the N-terminal arm is required for recruitment of TBC1D15 to the mitochondria, we expressed YFP-TBC1D15 and either pcDNA, FIS1 WT, or FIS1ΔN in WT or FIS1 CRISPR RPE cells. Cells were fixed and stained sequentially for Tom20, followed by FIS1, and imaged using confocal microscopy (Fig. 8). In WT RPE cells, YFP-TBC1D15 appeared largely cytosolic with a small mitochondrial fraction notable in medium and low expressing cells. Removal of endogenous FIS1 reduced this minor mitochondrial signal and resulted in a significant reduction in the Pearson’s correlation R value between YFP-TBC1D15 and Tom20. Expression of FIS1 WT resulted in profound mitochondrial recruitment of YFP-TBC1D15 (Fig. 8, *A* and *C*), consistent with earlier findings (38, 39). Expression of FIS1ΔN resulted in identical mitochondrial recruitment of YFP-TBC1D15, indicating the N-terminal arm is not required for TBC1D15 localization.

Strikingly, during the acquisition of these images, it became clear that the ΔN morphological phenotype was different upon TBC1D15 expression. In stark contrast to the DRP1 dataset aforementioned, coexpression of FIS1ΔN and YFP-TBC1D15 increased the proportion of highly fragmented mitochondria with an average MitoGraph connectivity score of 1.5 ± 0.3 (*versus* the DRP1 dataset ΔN value of 1.9 ± 0.6). These differences did not arise from differences in the probe used for segmentation (mitoYFP for the DRP1 dataset (Fig. 7*A*) and Tom20 for the TBC1D15 dataset (Fig. 8*A*)) since the MitoGraph connectivity scores are very similar between datasets with nearly identical averages between the TBC1D15 dataset (pcDNA: 2.3 ± 0.5 *versus* WT:1.8 ± 0.4; AVG ± STDEV) and the DRP1 dataset (pcDNA:2.3 ± 0.6 *versus* WT:1.7 ± 0.4). Despite the enhancement in mitochondrial fragmentation, the clumping phenotype was not altered by TBC1D15 expression. Mitochondria in cells expressing FIS1ΔN were highly fragmented and lacked the excessive clumping that we readily observe upon overexpression of FIS1 WT. This reduction in clumping resulted in more visibly fragmented/separated mitochondria and overall, likely contributed to an enhanced fragmentation profile for FIS1ΔN (Figs. S13 and S14). Plotting the Pearson’s R values for mitochondrial localization of YFP-TBC1D15 against the MitoGraph PHI score revealed that ΔN but not WT expression resulted in a reduction in PHI likely due to the difference in the clumping phenotypes (Fig. 8*D*).

Since YFP-TBC1D15 coexpression with FIS1ΔN partially rescued the ΔN defect in promoting mitochondrial fragmentation, we next queried if this coexpression resulted in stabilization of ΔN expression. We transfected mitoYFP or YFP-TBC1D15 and either pcDNA, FIS1 WT, or FIS1ΔN into WT and FIS1 CRISPR RPE cells. Cell lysates were probed for FIS1 and GFP by Western blot analysis. YFP-TBC1D15 coexpression resulted in more WT and slightly more FIS1ΔN expression relative to coexpression with mitoYFP, but ΔN expression remained substantially lower than WT (Figs. S15 and S16). We then limited the MitoGraph and colocalization analysis to cells expressing similar amounts of WT and FIS1ΔN and found comparable MitoGraph connectivity scores and Pearson’s correlation data for the YFP-TBC1D15 mitochondrial localization. Ultimately this expression analysis indicates the results described above were not due to the expression level differences between WT and FIS1ΔN nor was the enhanced fragmentation of FIS1ΔN due to restoring ΔN to WT expression levels.

These data indicate that the impaired fragmentation observed upon ΔN expression can be partially rescued by coexpression of TBC1D15. Expression of both WT and FIS1ΔN dramatically increased TBC1D15 localization (Fig. 8, *C* and *D*), indicating that FIS1 robustly drives mitochondrial recruitment of TBC1D15 and is independent of the FIS1 arm. The arm is, however, required for the clumping phenotype observed upon overexpression of FIS1 in a manner that TBC1D15 overexpression cannot rescue.

## Discussion

The role of FIS1 in DRP1-mediated mitochondrial fission is controversial as FIS1 deletion induces modest morphological changes in certain cell types (12, 18, 37, 62) including the FIS1 CRISPR RPEs generated for this study. Here, we focused on the FIS1 arm for two reasons. First, it appears to negatively regulate in DRP1 interactions in yeast (50, 51). Second, the arm is the major structural difference between FIS1 orthologs consistent with the potential for a regulatory role (Fig. 1, S1). Combining MD simulations (Fig. 3), NMR (Figure 4, Figure 5, Figure 6), and other biophysical analyses (Fig. 6) revealed the ability of the FIS1 arm to populate an IN conformation through intramolecular contacts with a conserved surface (Fig. 3*D*). In the mouse FIS1 structure, interactions between arm residues L5/L8 and TPR residues R44, K46, V79, and Y82 stabilize the IN conformation. NMR and MD analyses here support similar interactions in human FIS1. In yeast, this IN conformation is mediated by arm residues in a similar manner involving I85 and Y88 (orthologous to human V79/Y82). These yeast residues mediate binding with recombinant Dnm1p *in vitro* where arm deletion is necessary to observe Fis1p-Dnm1p binding. Perhaps counterintuitively, FIS1 arm deletion in yeast cells loses Dnm1p localization to mitochondria. Here, we find that human FIS1 arm deletion reduces DRP1 recruitment to mitochondria (Fig. 7), reduces fragmentation, and notably eliminates mitochondrial clumping raising the intriguing possibility that features of FIS1 activity are conserved between yeast and human. Deletion of the FIS1 arm also reduces mitochondrial fragmentation and clumping in HeLa and HMEC-1 cells (Figs. S8 and S9), indicating that this region of FIS1 is important in more than one human cell type. Curiously, this region is also deleted in FIS1 isoforms found in mice and worms. These data are consistent with an important regulatory role for the FIS1 arm across species.

In budding yeast, impaired fission and Dnm1p recruitment upon FIS1ΔN expression is overcome by overexpression of the fission adapter Mdv1p (53). While mammals have no known Mdv1p ortholog, we find here a similar effect in that coexpression of TBC1D15 with FIS1ΔN recovers mitochondrial fragmentation. The basis for this is unclear but is not due to enhancing FIS1 stability (Fig. S16), and Mdv1p only shares <10% sequence identity with TBC1D15 with no known role in mitophagy. It is curious that TBC1D15 coexpression only rescues the defect in fragmentation not clumping. Previously, mitochondrial clumping has been attributed to kinesin-mediated transport of mitochondria (63), but a role for TBC1D15 or FIS1 in these processes has not been reported.

Evidence for an important role for the human FIS1 arm is also suggested by previous work. Jofuku *et al*. investigated a rat Fis1 construct lacking the first 10 residues, which eliminates mitochondrial clumping found with WT (25). Yoon *et al.* found that expression of FIS1 lacking residues 1 to 32 was unable to fragment mitochondria (16). Subsequent coimmunoprecipitation experiments revealed increased interactions with DRP1 but only upon deletion of the first 20 or 31 residues (31). These data are consistent with the FIS1 arm governing DRP1 interactions and the loss of DRP1 localization in our experiments upon deletion of the arm (Fig. 7*C*). However, we cannot exclude that the morphological changes observed here are due to indirect effects on DRP1 or DRP1-independent means, especially given the strong correlation of TBC1D15 mitochondrial recruitment with fragmentation upon either WT or ΔN expression (Fig. S14*D*). These data suggest FIS1-dependent changes to mitochondrial morphology may be more reliant on TBC1D15, and possibly its partner GTPase Rab7a, than DRP1. Additionally, a FIS1 construct lacking the first 31 residues increased the interaction with profusion GTPases mitofusin 1, mitofusin 2, and optic atrophy 1 (Mfn1, Mfn2, OPA1), suggesting that the mechanism of FIS1-induced fragmentation was by way of inhibiting the fusion machinery (64). These results provide an alternative explanation in which the mitochondrial fragmentation observed here (Figure 7, Figure 8) may be due to fusion inhibition. Future studies examining the impact of FIS1 arm deletion (N-terminal residues 1–8) on direct and indirect interactions with TBC1D15/17, RAB7A, DRP1, MFN1, MFN2, and OPA1 will be informative.

We suspect that the FIS1 arm can play dual rules in governing FIS1 activity, where the arm interconverts between an IN and OUT conformation to modulate FIS1 activity. In this model, the arm IN conformation can either create a new binding surface or act in an autoinhibitory manner by preventing binding. In support of playing an autoinhibitory role, removal of residues 1 to 31 (helix 1) increases FIS1-binding affinity for multiple peptides identified from a peptide phage display screen (65). Thus, the arm OUT conformation may allow for binding, akin to recombinant yeast FIS1 (50) but also be necessary for DRP1 recruitment in cells once the FIS1 conserved surface is revealed. In this model, the arm plays a dual role by both preventing and enhancing DRP1 binding in a conformation-dependent manner. The interconversion of these conformations could be governed by cellular cues including changes in pH such as found for yeast FIS1 (59) or through posttranslational modifications. For instance, FIS1 is known to be phosphorylated on Ser-29 (66), which may influence the arm conformation and thus, FIS1 activity. Nonetheless, the link between FIS1 phosphorylation and arm conformational changes remains to be explored.

The idea of a disordered-to-ordered structural transition being important for activity is not unique to FIS1. Many proteins including other TPRs are influenced by disordered regions in a similar autoregulatory manner (67). For example, the TPR domain of kinesin-1 light chain is regulated by intramolecular interactions akin to the one proposed here for FIS1. In the kinesin-1 system, a distal disordered N-terminal region occludes a binding site on the TPR domain, which becomes displaced upon cargo binding (68). In fact, a survey of 1236 TPR domains shows that more than ∼30% to 48% are immediately flanked by regions of intrinsic disorder (Fig. S17). Thus, disordered tails in other TPR containing proteins might act in a regulatory manner akin to FIS1.

## Experimental procedures

### Protein expression and purification

Recombinant ^15^N-FIS1^1-125^ or ^15^N-FIS1^9-125^ (ΔN) were expressed using a pQE30 vector as a His_6_–Smt3–FIS1^1-125^ fusion protein in BL21(DE3) *Escherichia coli* carrying the pREP4 plasmid that leaves native residues after removal of the His_6_–Smt3 (yeast small ubiquitin-like modifier protein) expression tag as described (69, 70). FIS1 constructs were purified using nickel affinity and size-exclusion chromatography as described previously (70). Samples were buffer exchanged into the indicated buffer using Vivaspin 20 centrifugal concentrators (GE Healthcare) with a molecular weight cutoff of 3 kDa. For buffer exchanges, 15 to 20 ml of new buffer was added to concentrators, centrifuged at 3320*g*, and repeated at least five times. Protein samples were stored at 4 °C until data collection.

### NMR spectroscopy

NMR HSQC spectra were collected in 3 mm NMR tubes (Bruker) on a 14.1 T Bruker Avance II spectrometer equipped with a 5 mm TCI cryoprobe with a z-axis gradient. ^1^H, ^15^N HSQC experiments were collected on 300 μM ^15^N-FIS1 in one of three sample conditions (1): physiological pH (PHYS): 100 mM Hepes pH 7.4, 200 mM NaCl, 1 mM DTT, 0.02% (w/v) sodium azide, 10% D_2_O, 25 °C (2), OUT (condition used to solve 1PC2.pdb structure): 10 mM Tris acetate pH 5.5, 10% D_2_O, 32 °C, and (3) IN (condition used to solve 1IYG.pdb structure): 20 mM sodium phosphate pH 6.0, 100 mM NaCl, 1 mM DTT, 10% D_2_O, 25 °C. ^1^H,^15^N HSQC experiments were collected with eight scans consisting of 1024 (t_2_) × 300 (t_1_) complex points with acquisition times of 51.2 ms (^1^H) and 75.0 ms (^15^N). Spectra were processed using NMRPipe (71) *via* NMRBox (72), analyzed using CARA (https://cara.nmr.ch/) (73), and visualized using XEASY (74) and Adobe Illustrator (CS5 15.0.2). The ^15^N–FIS1 chemical shift differences were calculated between ^15^N–FIS1 in the physiological condition and previously published FIS1 1PC2.pdb sample conditions using in-house R scripts as described previously (70). For 1PC2.pdb analysis, FIS1 residues 125 to 152 were excluded since they have no equivalent residues for comparison against the FIS1 construct used in this study (residues 1–125). No chemical shift assignments are available for 1IYG.pdb, which was solved as part of the RIKEN structural genomics consortium and are not published. Thus comparisons such as that presented in Fig. 2 are not possible. Chemical shift assignments for FIS1^1–125^ in PHYS condition were previously assigned using standard triple resonance NMR and are deposited in the Biological Magnetic Resonance Data Bank with BMRB accession number 27904 (70, 75). Chemical shift assignments for FIS19 to 125 in PHYS conditions were assigned by analysis of ^15^N-edited NOESY experiment collected at 14.1 T with a contact time of 200 ms.

### hetNOE experiments

We collected ^1^H, ^15^N hetNOE experiments on 300 μM ^15^N-FIS1 in each of the three sample conditions described previously: physiological pH (PHYS), OUT (condition used to solve 1PC2.pdb structure), and IN (condition used to solve 1IYG.pdb structure). ^1^H, ^15^N hetNOE experiments consisted of 32 scans with 2048 and 512 complex points in ^1^H and ^15^N dimensions. ^1^H, ^15^N hetNOE spectra were split into the reference and NOE spectra in Topspin 3.5pl7 (Bruker) and then processed with NMRPipe (76). Processed spectra were imported into CARA (62), where each residue crosspeak was selected and integrated. All reference and NOE crosspeak intensities were imported into R (77) and analyzed using Tidyverse (78), broom (79), and readxl (80).

### NMR chemical shift perturbations

^1^H, ^15^N HSQC spectra were collected on 100 μM ^15^N-labeled FIS1^1-125^ and ^15^N-FIS1ΔN^9-125^ in 100 mM Hepes pH 7.4, 200 mM NaCl, 1 mM DTT, 0.02% (w/v) sodium azide, and 10% D_2_O, (physiological pH condition). Spectra were collected, processed, and analyzed as above. Chemical shift assignments for ^15^N-FIS1ΔN were made from analysis of 3D 15N-edited NOESY spectrum guided by published assignments FIS1^1-125^. After spectral processing and peak picking in CARA (73), chemical shifts were exported and imported into R for analysis. To visualize NMR chemical shift differences between the published 1PC2.pdb structure and the construct used in this study, we used a distribution density plot or kernel density plot, as a function of secondary structure. The kernel density plot is a way of estimating an unknown probability density function. For this, NMR chemical shifts from FIS1 residues in the arm (residues 1–8), loops, and helices (as defined by canonical ϕ, ψ values) were analyzed using *geom_density* function in R *tidyverse*, which calculates the kernel density of every data point x_i_ according to equation 1:

(1) $$f\left(x\right)=\frac{1}{hN}\sum_{i=1}^{N}K[\left(x-x_{i}\right)/h]$$

assuming a Gaussian distribution where *K()* is the kernel fucntion, *h* is the bandwidth, and *N* is the number of chemical shift values.

Chemical shift perturbations of FIS1 residues upon removal of the arm were calculated, as described previously (70), according to equation 2, and plotted in R as a function of residue number using Tidyverse and readxl. Then, chemical shift perturbations were displayed onto a structure of FIS1^1PC2^ in a gradient fashion, where white represents no chemical shift perturbation and red represents the greatest chemical shift perturbation. All protein images were rendered in PyMol (81).

(2) $$\Delta \delta \;Chemical\;Shift=\sqrt{\left({\left(5\Delta \delta_{H}\right)}^{2}+{\left(\Delta \delta_{NH}\right)}^{2}\right)}$$

where Δδ Chemical Shift = total chemical shift perturbation and Δδ_*H*_ and Δδ_*NH*_ represent amide proton and nitrogen chemical shifts differences, respectively.

### NMR spin relaxation data

FIS1 arm and protein dynamics were determined using the Lipari–Szabo model-free formalism. For this R_1_, R_2_, and hetNOE NMR spin relaxation experiments were collected at 298K on a 600 μM sample of ^15^N-FIS1^1-125^ using standard pulse sequences at 11.7 and 14.1 T. The delays for R_1_ (20, 60, 100, 200, 400, 600, 800, 1200 ms) and R_2_ (17.6, 35.2, 52.8, 88.0, 123.2, 158.4 ms) were collected in random order to minimize systematic errors with two (R1) or four (R2) time points recorded in duplicate for error analysis. Peak heights were extracted and analyzed using the NMR Series tool in CCPN NMR Analysis software (https://ccpn.ac.uk/software/version-2/) (82). Spin relaxation rates were determined by nonlinear least-squares optimization tool in NMR Analysis to fit data to a single exponential for each residue. The resulting data were analyzed using the model-free formalism (83) with the FAST-Modelfree (https://ursula.chem.yale.edu/∼lorialab/software.php) (84) and Modelfree 4.2 (https://comdnmr.nysbc.org/comd-nmr-dissem/comd-nmr-software/software/modelfree) (85) software. Fitting relies heavily on an appropriate model for the diffusion tensor, which was initially estimated using the program quadric diffusion that uses the spin relaxation data to compare isotropic, axial, and anisotropic diffusion models. For this, the human and mouse NMR structures of FIS1^1-125^ (1PC2 and 1IYG) were translated to the center of mass using PDBinertia, and the rotational correlation times were estimated from R_2_/R_1_ ratios at either magnetic field strength using r2r1_tm. Independent of the starting structure or field strength, an isotropic model was found to be the best fit to the data and was used in an iterative process with multiple replicates using different random seed values and starting structures to determine estimates of the generalized order parameter, (S^2^), internal motion (τ_e_), chemical exchange (R_ex_), and the overall rotational correlation time (τ_c_). S^2^ values were then imported into R and plotted as a function of residue number using Tidyverse and readxl.

### MD simulations

All MD simulations were performed using GROMACS (https://www.gromacs.org/) version release 2018 (86). All MD simulations used the Amber99SB force field with TIP3P water molecules. All simulations included a 140 mM KCl charge neutralization in a dodecahedron box, which extended ≥10 Å from the edge and were run at 298 K. All simulations used a 2.0 fs inner time step equilibrated with ensembles in which the number of particles, system volume, pressure, and temperature, were conserved. Production MD runs used particle mesh Ewald electrostatics, vdW interaction cutoff of 10 Å, Parrinello–Rahman pressure coupling, and V-rescale temperature coupling. Snapshots were saved every 10 ps. For each FIS1 starting structure—1PC2.pdb and human FIS1 model derived from 1IYG.pdb—non-native sequence (cloning artifacts) was removed. The sequences were also truncated to residue 125 to match the residue length of our experimental construct. Then, a 1000-ns simulation was performed starting from each PDB structure and repeated three times. RMSD, RMSF, and atom–atom distance calculations were computed with GROMACS and further analyzed and visualized in R using the tidyverse (78), Peptides (87), and readxl (80) packages. All protein structures were aligned by incremental combinatorial extension (88) and rendered in PyMol (81). A homology model of human FIS1 (hFIS1^IN^) derived from the mouse FIS1 structure (1IYG.pdb) was produced in PyMol with the mutagenesis wizard of the nonnative C-terminal residues S121D, P123L, S124V, and S125G and the following residues mouse to human substitutions: K15L, N16K, R19K, Q25K, E49D, and R53K. Note, these six conservative substitutions are the only differences between human and mouse FIS1 amino acid sequences and are not proximal to the FIS1 arm and conserved concave pocket of FIS1.

### Sparta+ MD chemical shift predictions

Sparta+ chemical shift predictions from MD simulations were performed as described previously (89). Waters and ions were removed from each MD trajectory (1000 ns) and snapshots from every 1 ns of the simulation were saved as individual PDB files. This resulted in each simulation consisting of 1000 conformational states used in Sparta+ chemical shift predictions. Each individual MD snapshot was then energy minimized using a 200 step steepest descent minimization with the Amber03 force field, which was selected due to having been previously shown to improve chemical shift predictions from multiple chemical shift prediction tools (89, 90).

Sparta+ (91) was then used to predict chemical shifts for each residue from each energy minimized MD snapshot. For ^1^H and ^15^N dimensions, the difference between FIS1^1-125^ chemical shifts (collected at physiological pH or PHYS sample condition) and Sparta+ chemical shift predictions from the FIS1 arm OUT (1PC2.pdb) and FIS1 arm IN (1IYG.pdb) conformations were then computed according to equation 3:

(3) $$\Delta \Delta \Delta \delta =\left|\delta_{OUT}-\delta_{PHYS}\right|-\left|\delta_{IN}-\delta_{PHYS}\right|$$

where ΔΔΔδ = total (^1^H or ^15^N) difference in chemical shift between the differences of FIS1^1-125^ at physiological pH (PHYS) and FIS1 arm OUT conformation (OUT), and FIS1^1-125^ at physiological pH (PHYS) and FIS1 arm IN conformation (IN); δ_OUT_ = Average Sparta+ chemical shift prediction from FIS1 arm OUT conformation (1PC2.pdb), δ_IN_ = Average Sparta+ chemical shift prediction from FIS1 arm IN conformation (1IYG.pdb), and δ_PHYS_ = FIS1^1-125^ experimentally measured chemical shifts measured at physiological pH. All data analysis and visualization of Sparta+ MD chemical shift predictions were performed in R using the following packages: tidyverse (78), broom (79), readxl (80), and gridExtra (92).

### Talos+ torsion angle predictions

Backbone torsion angles were predicted using Talos+ (57) FIS1^1-125^ chemical shifts measured at physiological pH (labeled as PHYS) and previously determined structures of FIS1 arm OUT conformation (1PC2.pdb, state 1, labeled as OUT PDB) and mouse FIS1 arm IN conformation (1IYG.pdb, state 1, labeled as IN PDB). All data analysis and visualization of Talos+ torsion angles were performed in R using tidyverse (78), broom (79), readxl (80), and gridExtra (92).

### Intrinsic tryptophan fluorescence

Intrinsic tryptophan fluorescence data of FIS1 or FIS1ΔN (10 μM) were collected on a PTI fluorimeter with excitation and emission slit widths of 4 and 6 nm, respectively. Protein samples were excited at 295 nm and emission spectra collected from 300 to 400 nm. Samples were placed in a Starna Cell 3–Q–10 quartz fluorimeter rectangular cell with a pathlength of 1 cm. Acrylamide quenching experiments were then performed under the same conditions using increasing amounts of acrylamide (0, 50, 100, 200, 300, 400, 500 mM) in 200 μl reactions diluted with 20 mM Hepes, pH 7.4, 175 mM NaCl, 1 mM DTT, and 0.02% NaN_3_. Reactions were incubated at room temperature (RT) for 30 min prior to spectra collection. Data were imported into R for analysis and visualization using Tidyverse and readxl. Emission spectra were buffer corrected to account for any background fluorescence from buffer components. The fluorescence at 341 nm of each protein alone divided by fluorescence in the presence of quenching agent (F_0_/F) was determined and plotted on the *y*-axis against the corresponding acrylamide concentration on the *x*-axis. Error bars represent SD of three technical replicates. The resulting data were fit to the Stern–Volmer equation F_0_/F = 1 + K_sv_∗[acrylamide]. The Stern–Volmer constant (K_sv_) ± SD was then calculated for FIS1 and FIS1ΔN.

### Thermal shift assay by NanoDSF

Protein unfolding was monitored at 330 nm and 350 nm using a Prometheus NT.48 (NanoTemper). FIS1 and FIS1ΔN were prepared at a final concentration of 25 μM in 100 mM Hepes, pH 7.4, 200 mM NaCl, 1 mM DTT, 0.02% NaN_3_. Approximately 10 μl per sample were loaded into Prometheus NT.48 Series nanoDSF high sensitivity capillaries (NanoTemper). A melting scan was performed using the Pr.ThermControl software (NanoTemper) with an excitation power of 100%, temperature range of 25 °C to 95 °C, and temperature ramp of 1 °C/min. The midpoint of the thermal unfolding curve (*T*_m_) was determined as the temperature corresponding to the maximum value of the first derivative of the 330 nm/350 nm fluorescence signal. Data were imported into R using readxl where box and whisker plots were generated using Tidyverse with *T*_m_ value represented on the *y*-axis and protein construct on the *x*-axis. Three biological replicates, each with three technical replicates, were used for *T*_m_ determination with error represented as SD.

### Cell culture

HMEC–1 cells (ATCC) were cultured in MCDB-131 supplemented with 10 ng/ml EGF, 1 μg/ml hydrocortisone, 10 mM glutamine, 10% fetal bovine serum, and 10 mM Hepes. Human RPE cells (RPE or ARPE–19, ATCC) were cultured in Dulbecco's modified Eagle's medium (DMEM)–F12 (Thermo Fisher Scientific) supplemented with 10% fetal bovine serum (Gemini). HeLa cells were cultured in DMEM (Thermo Fisher Scientific) supplemented with 1× nonessential amino acids, 2 mM glutamine, 1 mM sodium pyruvate, and 10 mM Hepes. See table of reagents in supporting information for full details of chemicals and suppliers.

### Transfection

Cells were either plated on a clean and sterilized No. 1.5 cover glass placed in a 6-well tissue culture dish using medium lacking antibiotics or in No. 1.5 glass bottom 24-well dishes (Cellvis). Approximately 24 h post plating, the cells were prepared for transfection (see table of reagents in supporting information for details). Plasmid DNA was added to Opti–MEM and briefly mixed by vortexing. The transfection reagent, Avalanche–Omni, was briefly vortexed and then added to the DNA:Opti–MEM mixture, immediately followed by vortexing for an additional 5 s. The complexes were incubated at RT for 15 min and added dropwise into each well. The cells were incubated overnight for 18 to 24 h and then processed for immunofluorescence.

### Immunofluorescence

Cells were prepared for immunofluorescence experiments either by following the methods outlined in (34) or optimized to reduce nonspecific binding and background speckling as described below. Once the cells achieved 70% to 80% confluency, the medium was aspirated and replaced with 4% paraformaldehyde (prewarmed to 37 °C) and incubated with gentle shaking at RT for 20 to 25 min (see table of reagents in supporting information for details). Fixative was removed and replaced with PBS. Following fixation, the cells were permeabilized by incubating with PBS/0.15% Triton X–100 for 15 min, followed by a brief wash in PBS, and incubation with blocking solution (0.3% bovine serum albumin/0.3% Triton X-100/PBS) for 1 h. Cells were then incubated overnight with primary antibody mix/5% normal goat serum/blocking solution, washed three times in PBS, incubated for 1 h with secondary antibody/blocking solution, and washed 2× in PBS/0.05% Tween-20 and once in PBS. The coverslips from 6-well plates were then rinsed in water, inverted, and mounted on glass slides in either p-phenylenediamine mounting medium (50 mM Tris pH 9.0, 45% glycerol (v/v) containing 2 mg/ml of the antifade reagent p–phenylenediamine) or Everbrite mounting media. Cells plated in 24-well plates were imaged in PBS. Note, to minimize antibody crossreactivity dual-labeling experiments from Figs. 7 and 8 were processed sequentially, first staining DRP1 or Tom20, followed by staining for FIS1.

### CRISPR/Cas

RPE cells were plated in a 6-well dish, and 24 h later, the cells were transfected with px462(v2) FIS1 Guide 1A and Guide 1B (FIS1 #1) or px462(v2) FIS1 Guide 2A and Guide 2B (FIS1 #2, see Fig. S6 or table of reagents in supporting information for more details). After 24 h, the medium was aspirated and fresh medium containing 2 μg/ml puromycin was added. The following day the media was again changed to fresh medium containing 2 μg/ml puromycin. Conditioned RPE media (50% fresh; 50% from confluent dish supernatant (centrifuged to remove floating cells)) was added at 72 h post transfection (note 48 h of puromycin treatment was sufficient to kill all cells in the untransfected condition). Once cells recovered from the puromycin treatment, they were expanded and froze down. Vials were thawed and grown in culture for several days prior to passaging into 96-well plates for clonal expansion (plated at densities of 1 and 2- cells/well). Note that the media used to plate these cells contained 25% conditioned media from a confluent matched plate. After approximately 3 weeks in culture, clones were moved into 24 well plates; once those wells were confluent the clone was split into 3-wells of 12-well plate. Once confluent, one well was collected for Western blot analysis, one well cryopreserved and the last well propagated. The pellet was washed once in PBS, repelleted, and stored at −20 °C.

### Western blot

Frozen cell pellets were thawed on ice, resuspended in radioimmunoprecipitation buffer containing protease inhibitor cocktail, incubated on ice 15 min, and centrifuged for 15 min at 14,000 rpm at 4 °C. The amount of total protein was quantified using a bicinchoninic acid assay. The sample was boiled in 1× Laemmli buffer, 10 to 15 μg of total protein was loaded on a 4% to 20% TGX gel (BioRad), transferred to a nitrocellulose membrane, blocked with 5% nonfat dry milk in TBST buffer (20 mM Tris, 150 mM NaCl, 0.1% Tween 20, pH 7.4), incubated overnight at 4 °C with anti-FIS1 primary antibody, washed three times in TBST, incubated for 1 h at 25 °C with anti-rabbit horseradish peroxidase secondary antibody, and the signal was detected using SuperSignal West Pico luminol reagent and visualized using Hyperfilm electrochemiluminescence or using the ChemiDoc MP Imaging System (Bio-Rad). Note prior to blocking, the membrane was briefly incubated with Ponceau S, rinsed in water, and imaged to observe total protein loaded.

### Image acquisition, colocalization, and intensity analysis

Cells were visualized using several different confocal microscopes (see reagent table for detailed information). For morphology counts, cells were visualized using a 60× oil objective and assessed by eye for the indicated morphology. Representative confocal images were acquired and processed using ImageJ2. For colocalization analysis, the ImageJ coloc2 plugin was used to calculate the Pearson’s correlation between endogenous DRP1 and mitoYFP or YFP-TBC1D15 and endogenous Tom20. An ImageJ macro was created to use regions of interest (ROIs) and single channel/single cell z-stack images generated from MitoGraph preprocessing (described later) for the coloc 2 analysis. Maximum intensity projection image stacks and ROIs from MitoGraph preprocessing were used to measure the mean intensity of FIS1 within the selected ROI region. R was used to compile the Pearson’s data and combine in a merged dataset with the MitoGraph metrics and intensity analysis. Box plots and ANOVA statistical calculations were also performed using R.

### MitoGraph analysis of mitochondrial morphology

#### Image preprocessing

Cells were imaged using a spinning disk confocal microscope, collecting the entire mitochondrial network at 0.3 micron z-slices and 0.11 μm/pixel resolution. Images were prepared for MitoGraph analysis by cropping individual cells containing the mitoYFP or Tom20 signal. To crop cells in batch mode, three separate ImageJ macros (see ref (34)) were used: one to split channels into separate folders; one to generate a stack of z-projections (GenFramesMaxProjs.ijm) to facilitate outlining cells and the other to crop single cells (CropCells.ijm) and save as individual single cell z-stack TIFF files. Cells containing mitochondrial network from adjacent cells are not selected for analysis.

#### MitoGraph segmentation and noise removal

The cropped TIFF files were processed using the following commands:

**mitoYFP segmentation:** MitoGraph -xy 0.11 -z 0.3 -adaptive 10 -path cells.

**Tom20 segmentation:** MitoGraph -xy 0.11 -z 0.3 -adaptive 10 -scales 1.5 2.0 6 -path cells.

The resulting PNG files were compiled using an ImageJ macro and screened for accurate mitochondrial segmentation. Some of the Visualization Toolkit (VTK) files were assessed for proper node assignment (see Fig. S10 for examples and (34) for potential troubleshooting assistance). All PNG images were screened for significant artifacts on the edge of the cropped cell or at the edge of the TIFF image. These can appear from partial mitochondria from adjacent cells or due to intensity drop-offs at the edge of the ROI of the PNG file. Previously, adding noise during the crop cell batch processing could prevent these artifacts; however, the added noise was often detected as mitochondrial connected components when using the adaptive thresholding, causing a significant increase in artifacts. Completely eliminating the added noise appeared to worsen the artifactual components, so the script was modified to fill the area surrounding the cropped cell with the minimum intensity from the ROI. Rather than to exclude images that in which artifactual components persisted, we modified our R-script to exclude 2-node, 1-edge connected components that were longer than 100 μm (the majority of legitimate 2-node, 1-edge connected components start at a length closer to 15–20 μm). We verified that the components removed were from images that contained artifactual components.

Low signal to noise immunofluorescence images can result in poor segmentation with MitoGraph and even images with average signal to noise ratios can be “noisy” and have faint non-mitochondrial speckling (such as anti-Tom20 or anti-FIS1 immunostained mitochondria). This can result in non-mitochondrial regions being segmented during image processing and thus detected as connected components, which are artifactual. Despite adjusting scales or adaptive thresholding to limit detecting noise, we noted artifactual connected components in our dataset with some values repeating thousands of times in a data set. We created a histogram binning all connected components by length and noted most of these repeating artifactual components were smaller than 1 μm (Figs. S11*B* and S13*B*) that gave rise to an artifactual "shoulder" on the histogram assuming a normal distribution for the data (Figs. S11*C* and S13*C*). Such artifactual repeating connected components were observed in multiple datasets. To remove these artifacts, a frequency table assuming a normal distribtuion was used to filter out highly repetitive connected components from the datasets. Filtering the dataset resulted in removal of the repeating connected components (Figs. S11*E*, S13*E*) and strikingly removed the shoulder from the width histogram, which appeared almost entirely due to the repeating connected components (Figs. S11*F*, S13*F*). We speculate these repeating connected components are due to random bright pixels and voxels being detected as connected components. The trend from the datasets looked similar before and after the filtering of the repeating connected components Figs. S11*G versus H*, S13 *G versus H*). All MitoGraph data presented in the main text and supplement have been filtered by removing all connected components that repeating more than 0.05% within the dataset. Note that mitoYFP, which is brighter and less noisy than anti-Tom20 immunostained mitochondria, had a smaller shoulder on the width histogram and a lower amount of repeating connected components (Figs. S11, *B* and *C versus* S13, *B* and *C*).

#### MitoGraph metrics

R scripts were generated to extract a variety of parameters from the GNET files, which contain node IDs and node-to-node distances (see ref (34) for visual representation of the parameters). MitoGraph metrics stem from these node-to-node distances, which can be from either an endpoint to a branch point, an endpoint to another endpoint for individual mitochondria or a branch point to branch point for highly interconnected networks. MitoGraph analysis provides several parameters derived from graph theory that describe the mitochondrial network (34, 61). Some parameters are normalized to total mitochondrial length to account for differences in cell size. ***Total connected components*** represent the number of connected mitochondrial components and are calculated by dividing total number of mitochondrial components by total length of all the mitochondria within that cell. Highly fragmented networks have an increase in connected components that derives from many small mitochondria, whereas highly interconnected networks would have lower numbers of connected components. ***PHI*** represents the fraction of the longest connected component relative to the length of the entire network; thus, PHI of approximately 1 would indicate an entirely fused network, whereas PHI closer to zero would indicate an entirely fragmented network. In graph theory, an “edge” is the distance between two connected nodes (defined later) and here is a measure of mitochondrial length between two nodes. The ***Average edge length*** is calculated from the total length of mitochondrial components divided by total number of edges. Average edge length can increase either due to decreased branching or longer individual mitochondria. ***Total edges*** are calculated by dividing total number of edges by total length of the mitochondrial components. Highly branched networks have more edges as do entirely fragmented networks. ***Total nodes*** are calculated by dividing total node number by total length of the mitochondrial components. Highly branched networks have more nodes as each branch point contains a node and each end contains a node. Entirely fragmented mitochondria also have more nodes. To help differentiate between a highly branched network *versus* shorter but less connected mitochondrial networks, nodes are further classified by whether they have only one neighbor (*free ends*) or whether they have three or four neighbors (*3-way/4-way junctions*). This is calculated by assessing the degree distribution, P(k), and gives the proportion of nodes with (k-1) neighbors. The average degree is calculated by the equation 4:

(4) $$\text{Avg Degree}={\text{sum}}_{\text{k}\left(\text{k}*\text{Pk}\right)}=\left(\text{FreeEnds}*1\right)+\left(3\text{way}*3\right)+\left(4\text{way}*4\right)$$

***MitoGraph Connectivity Score*** is calculated by the sum of the factors elevated in a highly fused state and divided by the sum of factors elevated in a highly fragmented state using equation 5:

(5) MitoGraph Connectivity Score = (PHI+AvgEdgeLength+AvgDegree)/(#NodeNorm+#EdgeNorm+#CCNorm)

Box plots and ANOVA statistical calculations were also performed using R. MitoGraph can be downloaded free of charge at https://github.com/vianamp/MitoGraph*.* R-scripts used for MitoGraph analysis are readily available at https://github.com/Hill-Lab/MitoGraph-Contrib-RScripts. MitoGraph v3.0 was optimized to run on a 556 Core Linux MPI cluster using a Singularity container, which is available free on github (https://github.com/mcw-rcc/mitograph/blob/master/Singularity). This is an Ubuntu 16.04 container with a slight modification to MitoGraph CMake files to allow a newer version of VTK. MitoGraph processed 100 images in less than 24 h with around 20 Gb of required memory.

## Data availability

All R scripts used for data analysis and visualization are available upon request and/or for download at https://github.com/Hill-Lab/. The majority of the data are contained within the article; raw data is available upon request.

## Supporting information

This article contains supporting information.

## Conflict of interest

R. B. H. and K. A. N. have financial interest in Cytegen, a company developing therapies to improve mitochondrial function. However, neither the research described herein was supported by Cytegen nor was in collaboration with the company. The authors declare that they have no conflicts of interest with the contents of this article.
