# Supplementary material for: Structural studies of human fission protein FIS1 reveal a dynamic region important for GTPase DRP1 recruitment and mitochondrial fission
Source: J Biol Chem. 2022 Oct 20;298(12):102620. doi: 10.1016/j.jbc.2022.102620 (PMC9747602; doi:10.1016/j.jbc.2022.102620)
Supplement: Supporting Information [file mmc3.zip › 2022_Egner_Supporting_Information_FINAL_oct.docx]

**­­­­­**

**Structural studies of human fission protein FIS1 reveal a dynamic region important for GTPase DRP1 recruitment and mitochondrial fission**

John M. Egner^1^, Kelsey A. Nolden^1^, Megan C. Harwig^1^, Ryan P. Bonate^1^, Jaime De Anda^2^, Maxx H. Tessmer^3^, Elizabeth L. Noey^4^, Ugochukwu K. Ihenacho^1^, Ziwen Liu^2^, Francis C. Peterson^1^, Gerard C.L. Wong^2^, Michael E. Widlansky^5^, & R. Blake Hill^1*^

**This PDF file includes:**

- **Supplemental Figures:**
  - **Figure S1:** *Sequence alignment of FIS1 homologs*
  - **Figure S2:** NMR chemical shifts of human FIS1 are not substantially altered in different sample conditions.
  - **Figure S3:** *MD simulation measurements of FIS1 for each starting structure and replicate*
  - **Figure S4:** *Comparison of FIS1 experimental and Sparta+ predicted chemical shifts from MD*
  - **Figure S5:** *NMR spin relaxation experiments of FIS1 used to determine generalized order parameter (S^2^) values*.
  - **Figure S6**. *FIS1 CRISPR knockout in RPE cells*.
  - **Figure S7.** *Overexpression of WT FIS1, but not ∆N, induces fragmentation of the mitochondrial network in FIS1 CRISPR RPE line #2.*
  - **Figure S8.** *Overexpression of WT FIS1, but not ∆N induces fragmentation and clumping of the mitochondrial network in HeLa cells*
  - **Figure S9.** *Overexpression of WT FIS1, but not ∆N induces fragmentation and clumping of the mitochondrial network in Hmec-1 cells.*
  - **Figure S10.** *MitoGraph segmentation examples*
  - **Figure S11**: *Individual mitoYFP MitoGraph measurement metrics for WT or FIS1 CRISPR knockout RPE cells*.
  - **Figure S12:** *FIS1 ∆N has lower fission activity despite expression level differences.*
  - **Figure S13:** *Individual Tom20 MitoGraph measurement metrics for WT or FIS1 CRISPR knockout RPE cells*.
  - **Figure S14:** *Correlation between several individual MitoGraph metrics and colocalization of DRP1/TBC1D15 with mitochondria.*
  - **Figure S15**: *Co-expression of FIS1 ∆N and TBC1D15 restores fission activity, but not WT expression levels.*
  - **Figure S16:** *YFP-TBC1D15 co-expression with FIS1 WT and ∆N leads to enhanced FIS1 expression.*
  - **Figure S17:** *Prediction of disordered tails in TPR containing proteins.*
- **Supplemental Movie descriptions:**
  - **Movie S1**: *MD trajectory of human FIS1 using starting structure 1PC2.pdb*.
  - **Movie S2**: *MD trajectory of human FIS1 using starting structure 1IYG.pdb*.
- **Table of Reagents**
- **References**

**Figure S1.** *FIS1 constructs referenced in the current study and* *sequence alignment of FIS1 homologs.* A. Fis1 constructs referenced in the current study. B. TOP, wild type human FIS1 sequence is shown. The cytoplasmic domain is residues 1-125 and the transmembrane domain is 126-152. In the current study, the human FIS1^1-125^ native sequence is used. The human FIS1 NMR structure (1PC2.pdb) sequence contains native residues 1-145 with 7 non-native residues. This construct was soluble despite the presence of the transmembrane domain. The mouse FIS1 NMR structure (1IYG.pdb) sequence contains native residues 1-120 with a 7 and 6 non-native residues on the N- and C-termini, respectively. Non-native residues are shown in red. BOTTOM, *Sequence alignment of FIS1 homologs.* Sequence alignments of full-length FIS1 from model research organisms (Human: *Q9Y3D6, Mouse: Q9CQ92, Zebra Fish (D.rerio): A0A0H4P4U5, Fruit Fly (D. melanogaster): B7YZT2, Flatworm (C. elegans): Q20291, Mouse-ear cress (A. thaliana): Q9M1J1, Yeast (S. cerevisae): P40515).* T-Coffee program with ClustalW alignment, and BLOSUM matrix was used for the alignment and Jalview for rendering. Letters were colored using a 20% amino acid conservation visibility. Amino acid conservation scores, from 0 (lowest) to *(highest), are shown below for each position. Depicted above the alignment are topological domains shared among all species. In green, is the N-terminal arm with minimal amino acid conservation; in purple are the tandem tetratricopeptide repeat (TPR) motifs, which show a high degree of conservation across all species. The 8 solved structures of FIS1 are: solution structure of human FIS1 (FIS1, PDB ID: 1PC2), solution structure of mouse FIS1 (mFIS1, PDB ID: 1IYG), crystal structure of FIS1 (PDB ID: 1NZN), solution structure of yeast FIS1 (yFIS1, PDB ID: 1Y8M), crystal structure of yFIS1 complexed with a fragment of yeast adapter protein Mdv1 (PDB ID: 2PQN), crystal structure of yFIS1 complexed with a fragment of yeast adapter protein Caf4 (PDB ID: 2PQR), crystal structure of yFIS1 (PDB ID: 3O48), and crystal structure of yFIS1 complexed with a fragment of yeast adapter protein Mdv1 containing a coiled coil domain (PDB ID: 3UUX). FIS1 sequence identity varies across homologs with the highest variation residing in helix 1, helix 6, and the FIS1 arm, which is the disordered N-terminal region before the start of helix 1. This difference is highlighted through structural alignments of FIS1 including helix 1 and 6 (Helix 1 – 6 RMSD comparisons: FIS1^1PC2^ – mFIS1^1IYG^: 1.2 Å, FIS1^1PC2^ – yFIS1^1Y8M^: 1.72 Å, mFIS1^1IYG^ – yFIS1^1Y8M^: 1.99 Å). FIS1 structural similarity becomes more evident when comparing the TPR core residues, comprised of helices 2 – 5 (TPR RMSD comparisons: FIS1^1PC2^ – mFIS1^1IYG^: 0.95 Å, FIS1^1PC2^ – yFIS1^1Y8M^: 1.04 Å, mFIS1^1IYG^ – yFIS1^1Y8M^: 1.13 Å). The RMSD variation between FIS1 structures is largest between yeast and mammalian FIS1 (human and mouse FIS1). Across all FIS1 homologs, it is the FIS1 arm that is most varied. All yFIS1 structures show the arm adopting the IN conformation (see Figure 1), which is formed by an intramolecular interaction with the conserved concave surface. Likewise, the mFIS1 arm adopts the IN conformation, while only sharing 28% sequence identity with yFIS1, even with the mFIS1 arm being 8 residues shorter than yFIS1. Note, the mFIS1 structure does contain a cloning artifact (GSSGSSG) on the N-terminus, although this does not impede the ability of the mFIS1 arm to form intramolecular contacts.

**Figure S2**. *NMR chemical shifts of human FIS1 are not substantially altered in different sample conditions*. Total ^1^H, ^15^N chemical shift perturbations for FIS1^1-125^ were determined and plotted between the following sample conditions: (A, B) physiological pH 7.4 and sample conditions used to solve solution structure of FIS1 (PDB ID: 1PC2, arm OUT conformation, 10 mM Tris Acetate pH 5.5, 10% D_2_O, 32 °C), (C, D) physiological pH 7.4 and sample conditions used to solve solution structure of mFIS1 isoform 1 (PDB ID: 1IYG, arm IN conformation, 20 mM sodium phosphate pH 6.0, 100 mM NaCl, 1 mM DTT, 10% D_2_O, 25 °C). The probability density distribution for the chemical shift perturbations in A, C are shown in B, D, respectively.

Ghjk

­

**Figure S3.** *MD simulation measurements across 3 replicates with FIS1 starting structures 1PC2 and h1IYG.* (A) C_α_ root mean square deviation (RMSD) changes from starting structures 1PC2 or h1IYG across each 1000 ns trajectory replicate. (B) The root mean square fluctuation (RMSF), a measure of sidechain flexibility, for each FIS1 residue across each 1000 ns trajectory replicate. (C) Atom–atom distances for indicated atoms during each MD simulation replicate. Atom pairs were selected by having one of each pair be located on the FIS1 arm or TPR core. Data from starting structures 1PC2 and h1IYG are colored in grey and cyan, respectively.

 **Figure S4**: *Comparison of experimental and MD simulation Sparta+ predicted chemical shifts of human FIS1.* Comparison of ^1^H and ^15^N FIS1^PHYS^ experimentally determined chemical shifts with average Sparta+ predicted chemical shifts from MD simulations (1000 ns, n = 3) using two FIS1 starting structures, referred to here as FIS1^1PC2^ and FIS1^h1IYG^. The FIS1^1PC2^ structure refers to a starting structure of model 1 of 1PC2.pdb in which the FIS1 arm is randomly oriented away from the TPR surface. The FIS1^h1IYG^ structure refers to a starting structure of a homology model using mouse FIS1 structure (1IYG.pdb) in which the FIS1 arm makes intimate contacts with the TPR surface. For each starting structure, MD trajectories were separated by every 1 ns resulting in 1,000 snapshots per simulation, which was then used for Sparta+ chemical shift predictions and each replicate averaged together for comparison against the experimentally measured FIS1 chemical shifts. (A, B) The differences between ^1^H and ^15^N FIS1 experimental and MD simulation Sparta+ predicted chemical shifts are shown for starting structure 1PC2. (C, D) The differences between ^1^H and ^15^N FIS1 experimental and MD simulation Sparta+ predicted chemical shifts are shown for starting structure 1PC2. (E, F) For 1H and 15N chemical shifts, the chemical shift differences of experimental and MD simulation Sparta+ predicted chemical shifts between starting structures is shown. If |^MD^δ_1pc2_ – ^Exp^δ_PHYS_| = 0, then the experimental chemical shift is in better agreement with an arm OUT conformation; whereas if |^MD^δ_h1IYG_ – ^Exp^δ_PHYS_| = 0, then the experimental chemical shift is in better agreement with an arm IN conformation. Therefore, if |^MD^δ_1pc2_ – ^Exp^δ_PHYS_| – |^MD^δ_h1IYG_ – ^Exp^δ_PHYS_|: is > 0, then the experimental chemical shift is in better agreement with an arm IN conformation; or is < 0, then the experimental chemical shift is in better agreement with an arm OUT conformation. The red dotted line indicates reported Sparta+ standard deviation prediction accuracy from benchmark experiments. Note that only two (1H) or three (15N) residues are confidently predicted and provide conflicting results. These residues may be ascribed to the FIS1 arm making slightly different contacts and/or conformations in the TPR concave pocket between each MD trajectory. This is reflected in the lower average atom–atom distance of Y76^CE1^:V4^CG2^ of h1IYG MD trajectories than the 1PC2 MD trajectories (**Figure S3D**). Alternatively, this may suggest illegitimate conformational spaces and motions being sampled due to force field insufficiencies or poor sampling coverage. Together, experimental FIS1^1–125^ chemical shift values measured at pH 7.4 are in good agreement with conformations sampled from both MD trajectories of different starting structures, suggesting FIS1^1–125^ is able to sample both arm IN and OUT conformations in solution. Residues are colored based on secondary structure.

z

**Figure S5.** *NMR spin relaxation experiments of FIS1 used to determine generalized order parameter (S^2^) values*. (A) spin-lattice relaxation rates, R_1_ (B) spin-spin relaxation rates, R_2_. (C) and steady-state heteronuclear NOE (I/I_0_) as a function of FIS1 residue number. Data were collected on 600 µM ^15^N-FIS1^1-125^ at 298K under PHYS conditions (100 mM HEPES pH 7.4, 200 mM NaCl, 1 mM DTT, 0.02% sodium azide, 10% D_2_O, 25 °C) at 11.7 T (500 MHz 1H) and (14.1 T (600 MHz 1H) as described in methods. We note that prior R2 measurements with the human 1PC2 construct show both decreased and increased values for arm residues Compared to helix 1 residues whereas only reduced values are found for the 1-125 construct here indicating that the construct length can influence arm dynamics.

**Figure S6**. *FIS1 CRISPR knockout in RPE cells*. (A) Table of guide RNAs used in to create the CRISPR knockout RPE cells. (B) WT or FIS1 CRISPR RPE cell lysates were probed for FIS1 while total protein input was observed via Ponceau S.

**Figure S7.** *Overexpression of WT FIS1, but not ∆N, induces fragmentation of the mitochondrial network in FIS1 CRISPR RPE line #2.* **(**A-B) FIS1 CRISPR #2 knockout RPE cells were transfected with mitoYFP and either pcDNA, pcDNA-FIS1 or pcDNA-∆N-FIS1, fixed and immunostained for FIS1 (Rb anti-FIS1; Proteintech) (A) Representative confocal images of anti-FIS1 and mitoYFP collected using a spinning disk confocal microscope (Nikon Ti2 CSU W1. (B) MitoGraph connectivity score was calculated by a ratio of pro–fission and pro–fusion MitoGraph metrics (see methods for more details). P-values were calculated by ANOVA followed by TUKEY post hoc analysis; p-values: * (p<0.05); ** (p<0.01); *** (p<0.001). n.s. = not significant. Scale bar represents 10µm.

**Figure S8.** *Overexpression of WT FIS1, but not ∆N induces fragmentation and clumping of the mitochondrial network in HeLa cells.* (A-B) HeLa cells were transfected with mitoYFP and either pcDNA, pcDNA-FIS1 or pcDNA-∆N-FIS1, fixed and immunostained for cytochrome c and imaged using a Nikon A1R confocal microscope. Note that FIS1 was not stained at this time due to lack of suitable antibody. We later identified a suitable FIS1 antibody (Proteintech) that even worked for endogenous FIS1 staining, whereas all other FIS1 antibodies that were previously tested either did not work at all or did not work for endogenous protein. (B) At least 200 cells were counted per condition in two independent experiments. The mitochondrial morphology was designated as either elongated, fragmented, clumped and fragmented or clumped and elongated (Mean ± SD). Scale bar represents 10µm.

**Figure S9.** *Overexpression of WT FIS1, but not ∆N induces fragmentation and clumping of the mitochondrial network in Hmec-1 cells.* (A-B) Hmec-1 cells were transfected with mitoYFP and either pcDNA, pcDNA-FIS1 or pcDNA-∆N-FIS1, fixed and immunostained for FIS1 and imaged using a spinning disk confocal microscope (Olympus Ix83 DSU). Note that the antibody used here to detect FIS1 (Ms anti-FIS1; Sigma) was sufficient to detect overexpressed, but ***not*** endogenous FIS1 (no signal under the endogenous scenario in the pcDNA samples). (B) At least 100 cells were counted per condition in three independent experiments. The mitochondrial morphology was designated as either elongated, fragmented or clumped (mean ± SD). P-values were calculated by ANOVA followed by TUKEY post hoc analysis. Scale bar represents 10µm.

**Figure S10.** *MitoGraph segmentation examples.* Representative images demonstrating the quality of MitoGraph segmentation. FIS1 #1 CRISPR knockout RPE cells were transfected with mitoYFP and either pcDNA, pcDNA-FIS1 WT or pcDNA-FIS1∆N, fixed, imaged using a spinning disk confocal microscope and cropped to single cells using ImageJ2 (ROI outline noted in yellow dashed lines; surrounding area padded with the minimum intensity of the image to prevent MitoGraph errors). The individual cell z-stack was processed with MitoGraph v3.0 using the following command: MitoGraph -xy 0.11 -z 0.3 -adaptive 10 -path cells. MitoGraph v3.0 output files demonstrating successful binary segmentation (PNG) and the accurate designation of nodes (VTK files). Nodes are depicted with white circles and are found at junction points and end points. Accurate node assignment is essential to all MitoGraph connected component analysis. Scale bar represents 10µm.

**Figure S11**: *Individual mitoYFP MitoGraph measurement metrics for WT or FIS1 CRISPR knockout RPE cells*. (A-C). Analysis of unfiltered connected components from the mitoYFP dataset (Figure 7). (A) Width versus length of connected components, where lighter blue indicates more nodes/more complex connected components. (B) Focused histogram analysis (binned at 0.001) of small connected components (less than 1.5µm). Note repeating connected components with thousands of repeating instances. (C) Width histogram (binned at 0.001) of all connected components, note shoulder (red arrow). (D-F) Connected components were processed to remove all connected components repeating more than 0.05% of the data set, note long rogue lines were also removed (see MitoGraph original paper). (D) Width versus Length of filtered connected components. (E) Focused histogram analysis (binned at 0.001) on small filtered connected components. Note removal of all connected components repeating more than 50 times. (F) Width histogram (binned at 0.001) of all filtered connected components, note absence of shoulder (red arrow), indicating the repeating connected components were contributing to this shoulder. These repeating connected components are likely due to bright noisy pixels or voxels that are being segmented and depicted as actual connected components. (G-H) MitoGraph parameter scores from unfiltered (G) or filtered (H) connected components from mitoYFP segmentation of WT or FIS1 CRISPR knockout RPE cells transfected with mitoYFP and either pcDNA (WT dark grey; FIS1 CRISPR light grey), pcDNA-FIS1 (blue) or pcDNA-∆N-FIS1 (red). See main text MitoGraph analysis of mitochondrial morphology section for details on each parameter. *all metrics noted with asterisk were normalized against the total length of the mitochondria to account for cell size differences.

**Figure S12:** *FIS1∆N has lower fission activity despite expression level differences.* (A-B) Western blot protein quantification (A) and densitometry analysis (B) of WT or FIS1 CRISPR RPE cells expressing pcDNA (V), pcDNA-FIS1 WT (WT) or pcDNA-FIS1-∆N (∆N). (A) RIPA buffer extracted cell lysates were probed for FIS1 and Tubulin while total protein input was observed via Ponceau S. A separate western blot was run (with a blank lane in the middle) to analyze DRP1 expression with total protein observed via the stain free dye. (B) Potential differences in FIS1 and DRP1 expression was measured using Image Lab software. (C-F) Data presented in Figure 7 was further analyzed for FIS1 or DRP1 intensity differences for the same region of interest used for MitoGraph and colocalization measurements. (C) Mean cellular fluorescence intensity for FIS1 confirms expression level differences between WT and ∆N FIS1. (D) MitoGraph Connectivity Score from both individual cells (small dots) and population means (large circles) were plotted against DRP1 mean intensity measurements for those cell ROIs. (E,F) MitoGraph Connectivity Score (E) or Pearson’s Correlation between DRP1 and mitoYFP (F) was plot against FIS1 mean cellular intensity. Any cells with FIS1 mean intensities above 2x standard deviation of ∆N FIS1’s mean intensity (noted in light grey boxes) were then filtered from the dataset. (G, H) The resulting filtered dataset contain cells only from the light grey box still demonstrated the FIS1 ∆N had reduced mitochondrial fission activity (G) and a reduction in DRP1 recruitment (H).

**Figure S13:** *Individual TOM20 MitoGraph measurement metrics for WT or FIS1 CRISPR knockout RPE cells*. (A-C). Analysis of unfiltered connected components from the TOM20 dataset (Fig. 8). (A) Width versus length of connected components, where lighter blue indicates more nodes/more complex connected components. (B) Focused histogram analysis (binned at 0.001) of small connected components (less than 1.5µm). Note repeating connected components with thousands of repeating instances. (C) Width histogram (binned at 0.001) of all connected components, note larger shoulder than mitoYFP segmentation (Fig. S11) (red arrow). (D-F) Connected components were processed to remove all connected components repeating more than 0.05% of the data set, note long rogue lines were also removed (see MitoGraph original paper). (D) Width versus Length of filtered connected components. (E) Focused histogram analysis (binned at 0.001) on small filtered connected components. Note removal of all connected components repeating more than 40 times. (F) Width histogram (binned at 0.001) of all filtered connected components, note absence of shoulder (red arrow) indicating the repeating connected components were contributing to this shoulder. These repeating connected components are likely due to bright noisy non-mitochondrial pixels or voxels that are being segmented and depicted as actual connected components. TOM20 had a larger shoulder than mitoYFP which is in line with the TOM20 immunostained mitochondria having lower S/N ratio than mitoYFP and more random non-mitochondrial speckles. (G-H) MitoGraph parameter scores from unfiltered (G) or filtered (H) connected components from TOM20 segmentation of WT or FIS1 CRISPR knockout RPE cells transfected with YFP-TBC1D15 and either pcDNA (grey), pcDNA-FIS1 (blue) or pcDNA-∆N-FIS1 (red). See main text MitoGraph analysis of mitochondrial morphology section for details on each parameter. *all metrics noted with asterisk were normalized against the total length of the mitochondria to account for cell size differences.

**Figure S14:** *Correlation between several individual MitoGraph metrics and colocalization of DRP1/TBC1D15 with mitochondria.* (A-C) Analysis of individual MitoGraph metrics from the DRP1/mitoYFP dataset shown in Figure 7. (A) DRP1 colocalization with MitoGraph Connectivity Score. Cells overexpressing FIS1 WT that were highly clumped/aggregated also a high degree of DRP1 colocalization to mitochondria. FIS1 ∆N does not readily clump mitochondria, nor does it lead to accumulation of DRP1. Increased DRP1 colocalization to mitochondria also correlates with a reduction in edge length (shorter mitochondria and shorter branches) (B) and also correlates with an overall reduction in total mitochondrial length (C). This is also likely reporting on the clumped/reduced mitochondrial numbers observed upon overexpression of FIS1 WT but not ∆N. (D-F) Analysis of individual MitoGraph metrics from the YFP-TBC1D15/TOM20 dataset shown in Figure 8. (D) Strong correlation between TBC1D15 mitochondrial localization and MitoGraph Connectiviy Score indicating that FIS1 WT and ∆N coexpression with TBC1D15 enhances mitochondrial fragmentation. (E-F) Strong correlation between enhanced TBC1D15 mitochondrial localization and reduced edge length and an overall reduction in total length of mitochondria.

Fdas

**Figure S15**. *Co-expression of FIS1 ∆N and TBC1D15 restores fission activity, but not WT expression levels.*

(A-E) Data presented in Figure 8 was further analyzed for FIS1 intensity differences for the same region of interest used for the MitoGraph and colocalization measurements. (A) Mean cellular fluorescence intensity for FIS1 confirms expression level differences between WT and ∆N FIS1. (B,C) MitoGraph Connectivity Score (B) or Pearson’s Correlation between YFP-TBC1D15 and Tom20 (D) was plot against FIS1 mean cellular intensity. Any cells with FIS1 mean intensities above 2x standard deviation of ∆N FIS1’s mean intensity (noted in light grey boxes) were filtered from the dataset. (C,E) The resulting filtered dataset contains cells only from the light grey box and demonstrates that FIS1 WT and ∆N had similar mitochondrial fission activities and both similarly recruited YFP-TBC1D15 to the mitochondria.

**Figure S16:** *YFP-TBC1D15 co-expression with FIS1 WT and ∆N leads to enhanced FIS1 expression.* (A-B) Western blot protein quantification (A) and densitometry analysis (B) of WT or FIS1 CRISPR RPE cells expressing mitoYFP or YFP-TBC1D15 and pcDNA (V), pcDNA-FIS1 WT (WT) or pcDNA-FIS1-∆N (∆N). RIPA buffer extracted cell lysates were probed for FIS1 or GFP while total protein input was observed via Ponceau S. The same image was adjusted to use the entire histogram (0-65535; High) to illuminate differences in the high intensity bands; reduced by over 80% (0-12000; Medium) to illuminate when FIS1 ∆N was starting to be detected in the YFP-TBC1D15 samples (but not mitoYFP samples) or reduced by over 95% (0-2000; Low) to illuminate endogenous FIS1 and ∆N expression profiles. Differences in FIS1 expression was measured using Image Lab software and plot grouping all pcDNA, FIS1 WT or FIS1 ∆N samples together. Note elevation in FIS1 signal in the presence of YFP-TBC1D15 (closed circles/triangles) versus in the presence of mitoYFP (open circles/triangles).

**Figure S17:** *Prediction of disordered tails in TPR containing proteins.* Left panel, proportion of intrinsically disordered regions in a dataset of 637 N-terminal and 637 C-terminal sequences adjacent to tetratricopeptide repeats (TPRs) domains from a set of 637 TPR-domain containing proteins; the general proportion of intrinsically disordered scores in the full proteins (one score per amino acid) is shown as reference distribution of scores from the sampling set. A 0.30 fraction of the N-terminals and 0.48 of the C-terminals were characterized as being intrinsically disordered (prediction score > 0.5). Overall the proteins used in this analysis contained a 0.15 fraction of intrinsically disordered regions; hence, intrinsically disordered regions are not common in TPR containing proteins making the fraction of these intrinsically disordered N- and C-terminals unique in the vicinity of TPRs/TPR-domains. Right panel, Prediction of intrinsically disordered regions in FIS1; the location of secondary structure α-helices have been annotated for references using 3D structure file PDB:1PC2, and annotated transmembrane helix in UniProtKB: Q9Y3D6. In agreement with the solved 3D structure of FIS1, the IUPred3 predictor identified the N- and C-terminal tails to be intrinsically disordered (0.60 score), while the rest of the peptide has secondary structure (<0.5 score). For this analysis, 1055 sequences containing annotated TPRs were downloaded from UniProtKB/Swiss-Prot at the time of this study. TPRs are commonly adjacent 2-8 repeats creating a TPR domain; for this analysis only proteins containing a single TPR domain of 2-8 repeats were used, n=637. The TPR domains are flanked at their N- and C-termini by a ‘capping helix’ that is not part of the TPR repeat sequence and to account for this 17 amino acids preceding (N-terminal) and following (C-terminal) the TPR domains was excluded. To assess if the TPRs were capped by a disordered sequence, the full sequences were scored using IUPred3, an intrinsically disordered protein predictor using a window of 11 residues to identify intrinsically disordered amino acid sequences embedded in the proteins. We used the ‘short disorder’ option available in IUPred3, since we were interested in small sequential neighborhood of residues (<30 aa). The predictor outputs a score per amino acid position based on the amino acid sequence in the vicinity (windowed scoring). Since the IUPred3 output score per amino acid is influenced by the sequence vicinity and TPR are inherently highly α-helical, we allowed a spacer of 6 amino acids not used to calculate the terminal’s score, i.e., only 5 most distal amino acids were used. The intrinsically disordered score for each terminal was calculated as a mean value of the window scores.

**Movie S1:** *MD trajectory of human FIS1 using starting structure 1PC2.pdb*. Human FIS1 conformations were sampled by 100 ns MD simulations. The MD trajectory is shown for the initial starting structure PDB ID: 1PC2. Movie was rendered in PyMol.

**Movie S2:** *MD trajectory of human FIS1 using starting structure 1IYG.pdb*. Human FIS1 conformations were sampled by 100 ns MD simulations. The MD trajectory is shown for the initial starting structure PDB ID: 1IYG. Movie was rendered in PyMol.

**Table of Reagents**:

List of reagents used in this study. Source and additional notes are included as needed.

| **Item** | **Source** | **Notes** |
| --- | --- | --- |
| ***Chemicals*** | | |
| Amersham Hyperfilm ECL | GE Healthcare |  |
| ^15^N ammonium chloride | Cambridge Isotopes Laboratories |  |
| Avalanche-Omni | EZ Biosystems | • followed manufacturer protocol see cell lines below for line specific ratios used |
| DMEM-F12 | Thermo Fisher Scientific |  |
| EGF | Gibco; Thermo Fisher Scientific |  |
| FBS | Gemini |  |
| Glutamine | Gibco; Thermo Fisher Scientific |  |
| HEPES | Sigma |  |
| 1 M HEPES solution | Thermo Fisher Scientific |  |
| Hydrocortisone | Sigma |  |
| Laemelli Buffer 5x | Hill Lab | • 5X: 0.225 M Tris pH 6.8; 50% Glycerol; 5% SDS; 0.05 % Bromophenol Blue • 2-mercaptoethanol added fresh at 10% final volume in preparing samples |
| MCDB-131 media | Thermo Fisher Scientific |  |
| NEAA | Thermo Fisher Scientific |  |
| nonfat dry milk | Apex |  |
| p-phenylenediamine | Sigma |  |
| paraformaldehyde | Electron Microscopy Sciences | use at 4% in 1x PBS |
| Peirce BCA protein assay kit | Thermo Scientific |  |
| Ponceau S | Sigma |  |
| Protease Inhibitor Cocktail (EDTA free) | Roche |  |
| puromycin | Gibco; Thermo Fisher Scientific | use at 2µg/mL |
| RIPA Buffer | Pierce - ThermoFisher |  |
| sodium phosphate | Sigma |  |
| SuperSignal West Pico Luminol | Thermo Scientific |  |
| TBST | Hill Lab | • 25 mM Potassium Chloride; 140 mM NaCl; 0.05% Tween 20; pH 7.4 |
| Tris Acetate | Sigma |  |
| ***Cells*** | | |
| BL21 (DE3) pREP4 E. coli strain | Volkman lab | • KAN resistant |
| HeLa | Youle Lab | • plated 200,000 cells / 6-well  • Transfection condition:  - Total DNA: Avalanche (1.25µg:1µL)  - Amount FIS1: mitoYFP (1µg:0.25µg) |
| RPE (retinal pigmented epithelium) | ARPE-19 (ATCC CRL-2302) | • plated 150,000 cells / 6-well • morphology experiments transfection conditions:  - Total DNA: Avalanche (1.25µg:1µL)  - Amount FIS1: mitoYFP (0.6µg:0.2µg)  - Amount of pcDNA 0.45 µg  • CRISPR experiments transfection conditions:  - Total DNA: Avalanche (2.5µg:3.5µL)  - Amount px462 (1.25µg each) |
| Hmec-1 (human microvascular endothelial cells) | ATCC CRL-3243 | • plated 175,000 cells / 6-well • Transfection condition:  - Total DNA: Avalanche (2.5µg:3.5µL)  - Amount FIS1: mitoYFP (0.75µg:0.75µg)  - Amount of pcDNA: 1µg |
| ***Antibodies*** | | |
| Anti-TOM20 (Rb) | Santa Cruz; sc-11415 (discontinued) | • Immunostaining: 1:500 (2 hr @ RT) |
| Anti-TOM20 (Ms) | Santa Cruz; sc-17764 | • Immunostaining: 1:100 (o/n @ 4˚C) |
| Anti-Cyt c (Ms ) | BD Pharmingen; 556432 | • Immunostaining: 1:500 (2 hr @ RT) |
| Anti-FIS1 (Ms) | Sigma; WHOO51024M1 | • Immunostaining: 1:500 (2 hr @ RT) • works for overexpressed FIS1 immunostaining |
| Anti-DRP1 (Ms) | BD; 611112 | • Immunostaining: 1:100 (o/n @ 4˚C) |
| Anti-FIS1 (Rb) | RB; Proteintech; 10956-1-AP | • Immunostaining: 1:500 (o/n @ 4˚C) • Western Blot: 1:500 - 1:1000 (o/n @ 4˚C) * works for overexpressed and endogenous immunostaining and western blot analysis |
| Alexa Fluor 568 goat anti-mouse IgG (H+L) | Life Technologies (A11004) | • Immunostaining: 1:500 (1 hr @ RT) |
| Alexa Fluor 405 goat anti-rabbit IgG (H+L) | Life Technologies (A31556) |  |
| Alexa Fluor plus 647 goat anti-mouse IgG (H+L); highly cross absorbed | Invitrogen (A32728) |  |
| F(ab’)2 Goat anti-Mouse IgG (H+L); cross-absorbed | Invitrogen (A48289TR) |  |
| Goat anti-Rabbit IgG (H+L) Cross-Adsorbed Secondary Antibody, HRP | Thermo Fisher Scientific  (G21234) | • Western Blot: 1:1000 (1-2 hr @ RT) |
| Goat anti-Mouse IgG (H+L) Cross-Adsorbed Secondary Antibody, HRP | Thermo Fisher Scientific (G-21040) | • Western Blot: 1:1000 (1-2 hr @ RT) |
| ***Plasmids*** | | |
| pQE30 Smt3 His6 FIS1^1-125^ | Hill & Volkman lab | • AMP resistant |
| pQE30 Smt3 His6  FIS1^9-125^ | Hill & Volkman lab | • AMP resistant |
| pcDNA 3.1 (-) | Thermo Fisher | • AMP resistant |
| pcDNA FIS1 WT | Hill Lab | • pcDNA 3.1 (+) • full length FIS1 • cloned 5' HindIII – 3' BamHI • FWD 5’ – 3’ primer: ACGTG AAGCTT ca ATGGAGGCCGTGCTGAAC • REV 5’– 3’ primer: ACGTG GGATCC TCAGGATTTGGACTTGGAC • AMP resistant |
| pcDNA ∆N FIS1 WT | Hill Lab | • pcDNA 3.1 (+) * hFis ∆N8, includes start methionine  • cloned 5' HindIII – 3' BamHI • FWD 5’ – 3’ primer: ACGTG AAGCTT ca atg GTGTCTGTGGAGGACCTGC • REV 5’ – 3’ primer: ACGTG GGATCC TCAGGATTTGGACTTGGAC • AMP resistant |
| mitoYFP | BD Bioscience | • KAN resistant |
| YFP-TBC1D15 | Youle Lab | • isoform 3  • KAN resistant |
| px462_FIS1_Guide1A | Hill Lab | • pSpCas9n(BB)-2A-Puro (PX462) V2.0 was a gift from Feng Zhang (Addgene plasmid # 62987; http://n2t.net/addgene:62987; RRID: Addgene_62987) • targets FIS1 exon 4: CATTGTACTTGCTCCGCACCAGG (score 81; pair score 72) • AMP • 5' enzyme: BbsI |
| px462_FIS1_Guide1B | Hill Lab | • pSpCas9n(BB)-2A-Puro (PX462) V2.0 was a gift from Feng Zhang (Addgene plasmid # 62987; http://n2t.net/addgene:62987; RRID: Addgene_62987) • targets FIS1 exon 4: TAAAGGCATCGTGCTGCTCGAGG (score 89; pair score 72) • AMP • 5' enzyme: BbsI |
| px462_FIS1_Guide2A | Hill Lab | • pSpCas9n(BB)-2A-Puro (PX462) V2.0 was a gift from Feng Zhang (Addgene plasmid # 62987; http://n2t.net/addgene:62987; RRID: Addgene_62987) • targets FIS1 exon 4: GTACTCAAACTGCGTGCTCTTGG (score 89; pair score 72) • AMP • 5' enzyme: BbsI |
| px462_FIS1_Guide2B | Hill Lab | • pSpCas9n(BB)-2A-Puro (PX462) V2.0 was a gift from Feng Zhang (Addgene plasmid # 62987; http://n2t.net/addgene:62987; RRID: Addgene_62987) • targets FIS1 exon 4: TACAATGATGACATCCGTAAAGG (score 90; pair score 72) • AMP • 5' enzyme: BbsI |
| ***Imaging systems*** | | |
| Nikon A1R | CVC | • HeLa experiment • Confocal microscope  • 0.125 µm/pixel resolution • 60x oil objective (PlanApo VC 1.4 NA) |
| Olympus Ix83 DSU | Marchese Lab | • Hmec-1 experiment • spinning disk confocal (Olympus Ix83 DSU) microscope (DSU in) • Hamamatsu ImagEM X2 EM-CCD Digital Camera (C9100-23B) • 0.2 micron z-slices; 0.16 µm/pixel resolution  • Cells were imaged using a 100x oil objective (UPlanSApo 1.4 NA). |
| Nikon Ti2 CSU W1 | Hill Lab & Hudson Lab | • RPE experiments • Nikon Eclipse Ti2-E microscope • Yokogawa confocal scanner unit (CSU-W1) (50 micron pinhole) • Hamamatsu ORCA-Flash4.0 V3 sCMOS Camera  • 0.3 micron z-slices; 0.11 µm/pixel resolution • Cells imaged using a 60 x oil objective (Plan Apo; 1.40 NA) |
| BioRad ChemiDoc MP Imaging System | Terhune Lab |  |
